# Multifocal organoids reveal clonal associations between synchronous intestinal tumors with pervasive heterogeneous drug responses

**DOI:** 10.1038/s41525-022-00313-0

**Published:** 2022-07-19

**Authors:** Nahyun Jeong, Soon-Chan Kim, Ji Won Park, Seul Gi Park, Ki-Hoan Nam, Ja Oh Lee, Young-Kyoung Shin, Jeong Mo Bae, Seung-Yong Jeong, Min Jung Kim, Ja-Lok Ku

**Affiliations:** 1 Korean Cell Line Bank, Laboratory of Cell Biology, Cancer Research Institute, Seoul National University College of Medicine, Seoul, 03080 Korea; 2 Cancer Research Institute, Seoul National University, Seoul, 03080 Korea; 3 Department of Biomedical Sciences, Seoul National University College of Medicine, Seoul, 03080 Korea; 4 Ischemic/Hypoxic Disease Institute, Seoul National University College of Medicine, Seoul, 03080 Korea; 5 Department of Surgery, Seoul National University College of Medicine, Seoul, 03080 Korea; 6 Division of Colorectal Surgery, Department of Surgery, Seoul National University Hospital, Seoul, 03080 Korea; 7 Laboratory Animal Resource Center, KRIBB, Chungbuk, 28116 Korea; 8 Department of Pathology, Seoul National University College of Medicine, Seoul, 03080 Korea

**Keywords:** Cancer genomics, Tumour heterogeneity, Cancer models

## Abstract

Multifocal colorectal cancer (CRC) comprises both clonally independent primary tumors caused by inherited predisposition and clonally related tumors mainly due to intraluminal spreading along an intact basement membrane. The distinction between these multifocal CRCs is essential because therapeutic strategies vary according to the clonal association of multiple tumor masses. Here, we report one unique case of synchronous intestinal cancer (SIC) with tumors occurring along the entire bowel tract, including the small intestine. We established six patient-derived organoids (PDOs), and patient-derived cell lines (PDCs) from each site of the SIC, which were subjected to extensive genomic, transcriptomic, and epigenomic sequencing. We also estimated the drug responses of each multifocal SIC to 25 clinically relevant therapeutic compounds to validate how the clinically actionable alternations between SICs were associated with drug sensitivity. Our data demonstrated distinct clonal associations across different organs, which were consistently supported by multi-omics analysis, as well as the accordant responses to various therapeutic compounds. Our results indicated the imminent drawback of a single tumor-based diagnosis of multifocal CRC and suggested the necessity of an in-depth molecular analysis of all tumor regions to avoid unexpected resistance to the currently available targeted therapies.

## Introduction

About 2–5% of colorectal cancer (CRC) cases develop multifocal tumor masses, which are classified as either synchronous or metachronous CRC. They are traditionally considered as multiple independent primary CRCs based on clinicopathologic distinctions such as the presence of the histologic growth pattern and a dysplastic precursor^1,2^. Previous studies have reported that known predisposing factors such as chromosomal instability (CIN), microsatellite instability (MSI) status, and DNA methylation account for only 10% of multifocal CRC cases. Although the clinicopathologic evaluation and a few genetic predispositions verified the intertumoral heterogeneity of multifocal CRCs, the clonal association among multiple tumors with respect to molecular characteristics remains unexplained. Only a few recent studies using multiregional sequencing approaches have indicated that the multifocal CRCs are not entirely independent of each other but clonally related in terms of mutational profiles and DNA copy numbers^3,4^. Nevertheless, the clonal relationship of multifocal CRCs at transcriptomic and epigenomic levels has not been thoroughly investigated, and more importantly, its implication with clinically applicable therapeutic compounds was not determined. From this viewpoint, the unique ability of organoid technology to construct clonal associations by establishing independent organoids from multifocal CRCs is valuable for simultaneously assessing both multi-layered molecular clonality and its impact on various drug responses.

Here, we report one unique case of synchronous intestinal cancers (SICs) occurring along the entire bowel tract including the small intestine ascending colon, transverse colon, descending colon, and rectum. We established six patient-derived organoids (PDOs), and patient-derived cell lines (PDCs) from each site of the SIC, which retained major histological features of original tumors. They were then subjected to extensive genomic, transcriptomic, and epigenomic sequencings. We also estimated drug responses of each multifocal SICs to 25 clinically relevant compounds in order to validate how the clonal association between SICs interlinked to the drug sensitivities. We detected unexpected clonal associations transcending across different organs among sub-regional tumors and their derivatives, which was consistently supported by multi-omics analysis as well as accordant responses to multiple compounds. Our study enabled us to link interactions between clinically actionable biomarkers of multifocal SICs in various molecular layers, which help predict heterogeneous responses of multifocal SICs to currently available targeted therapies.

## Results

### Multi-regional intestinal organoids and cell lines from a single patient recapitulated histological features of original tumors

**Fig. 1:**
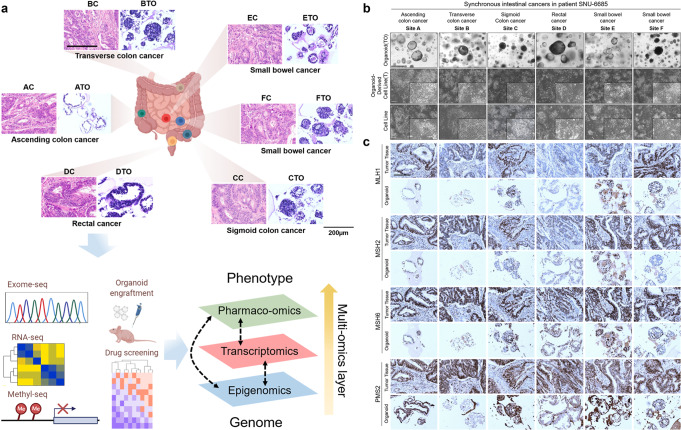
Histopathological characterization of SIC patient-derived organoids and cell lines. See also Supplementary Fig. 1 and Table 1**. a** Overall study design. The cartoons representing gut, exome-seq, RNA-seq, methyl-seq, organoid engraftment, drug screening, and arrows were illustrated by BioRender. The H&E slides of multifocal tumor tissues and organoids as well as the images representing multi-omics layers were original pictures created by the authors. We successfully generated 6 PDOs and 12 PDCs (6 PDCs and 6 PDO culture-derived PDC cultures) corresponding to one synchronous intestinal cancer (SIC) patient. We compared multi-omics data from all sub-regional derivatives and analyzed heterogeneous factors that might affect drug responses, Scale bar = 200 μm. **b** The morphology of SIC organoids and cell lines derived from the patient SNU-6685. All PDOs had mixed morphology and retained both the inter-tumor and intra-patient heterogeneity. Established cell lines were composed of relatively uniformed clones compared to organoids, cellular structures were comprised of mono-layered epithelial cells displaying polygonal morphology and cell aggregates were formed in all of PDCs and PDOCs. Scale bar = 500 μm and *insets* are ×200. **c** Organoids resemble primary tumor epithelium in histological features. The protein expressions of MMR genes include MutL Homolog 1 (*MLH1*), MutS homolog 2 (*MSH2*), MutS Homolog 6 (*MSH6*), and PMS1 Homolog 2 (*PMS2*) in PDOs resembled their corresponding tumors, Scale bar = 200 μm.

We successfully established patient-derived organoids (PDOs), cell lines (PDCs), and organoid-derived cell lines (PDOC) from each of six different sites of one synchronous intestinal tumor, resulting in 18 preclinical in vitro models retaining histological and molecular features of the original tumor. Each unique site of the SIC was designated as A–F in alphabetical order before in vitro cultivation. Successively, we performed genomic, transcriptomic, and epigenetic sequencing analysis as well as drug screening using a panel of 25 selected drugs. This enabled a comprehensive interpretation of clonal relationships among multiple tumors along the bowel tract as well as an interactive association between diverse omics, reflecting heterogeneous drug responses among multi-site clones. The overall study design is shown in Fig. 1a. The cartoon was created with BioRender.com. The patient had neither a family history of cancer nor inflammatory bowel disease, which are commonly found in multifocal CRC^3^. He did not show clinical features indicating polyposis syndromes such as oral mucosal pigmentation or other gastrointestinal polyps. He had liver, lung, and peritoneal metastases, and underwent right hemicolectomy, Hartmann’s operation, and small bowel segmental resections because he had obstructive symptoms and the liver metastases were not extensive. After surgery, he received 4 cycles of FOLFOX with Bevacizumab and the response was stable disease. However, after 4 cycles, despite the size of liver metastases being decreased, lung metastases were progressive. The regimen was changed to FOLFIRI with Bevacizumab, but the response was poor after 4 cycles of chemotherapy. The patient expired 1 year after surgery. Clinicopathological information including the six different locations of the SIC is summarized in Table 1 and Supplementary Data 1a.

**Table 1:** Clinicopathological characteristics of SNU-6685 patient.

Patient ID	Sex/Age	Sample name	Pathologic stage (AJCC 8th)			Location	Tumor size (cm^3^)	Pathological diagnosis	Invasion	Lymph node
			pT	pN	pM					Metastasis
SNU-6685	M/61	AC	4a	2b	1c^a^	Ascending colon	4.5 × 3.7 × 2.1	Adenocarcinoma, ulceroinfiltrative	LI^b^, VI^c^, PNI^d^	O
		BC	4a			Transverse colon	2.5 × 2.4 × 1.8	Adenocarcinoma, ulceroinfiltrative	LI	X
		CC	3c			Sigmoid colon	4.4 × 3.8 × 2.4	Adenocarcinoma, ulceroinfiltrative	LI, PNI	X
		DC	3c			Rectum	4.8 × 3.8 × 3.7	Adenocarcinoma, ulceroinfiltrative	LI, VI, PNI	X
		EC	3	1		Small bowel	5.7 × 3.5 × 1.7	Adenocarcinoma, annular constricting	LI, PNI	O
		FC	3			Small bowel	3.3 × 2.5 × 0.6	Adenocarcinoma, annular constricting	PNI	X

^a^Indicates omental metastasis

^b^LI indicates lymphatic invasion.

^c^VI indicates venous invasion.

^d^PNI indicates perineural invasion.

Six tumors were pathologically proven adenocarcinoma and pre-existing adenoma was not identified. Tumor deposit was present in carcinomas resected from ascending colon to the rectum (A–D). Short tandem repeat (STR) profiling validated that all derivatives shared matched loci without cross-contamination (Supplementary Data 1b). Established PDOs varied in morphology (e.g., spheroidal, asymmetric, and loose aggregated morphologies) (Fig. 1b; Supplementary Fig. 1a). Matched PDCs and PDOCs grew as monolayers of substrate-adherent cells displaying polygonal and spindle morphologies. The majority of PDCs formed adherent aggregates. Hematoxylin-eosin (H&E) staining of paraffin sections from each site of the tumor and corresponding PDOs displayed their histopathological features, showing moderately differentiated structures compared to their matched normal tissues (Supplementary Fig. 1a). There was a distinct morphological heterogeneity among multi-site PDOs (Fig. 1b). For instance, organoids derived from A, D, and E sites mainly exhibited thin-walled cystic structures whereas organoids from B, C, and F sites showed a loosely aggregated and compact form (Fig. 1a). In contrast, both PDCs and PDOCs displayed uniformed cellular structures with a multi-layered polygonal morphology (Fig. 1b). This suggests that organoid culture can better retain the morphological heterogeneity of the original tumor.

We then performed immunohistochemistry (IHC) of various tumor markers including mismatch repair (MMR) genes such as MutL homolog 1 (*MLH1*), MutS Homolog 2 (*MSH2*), MutS Homolog 6 (*MSH6*), and PMS1 Homolog 2 (*PMS2*) in order to confirm the correspondence of expressional patterns between PDOs and matched tumors. IHC revealed that PDOs recapitulated the heterogeneous distribution as well as intensities of such markers (Fig. 1c). PDOs also analogously expressed caudal type homeobox 2 (*CDX2*), nuclear β-catenin (*CTNNB1*), KI-67 (*MKI67*), and SMAD Family Member 4 (*SMAD4*) of corresponding tumors (Supplementary Fig. 1b). β-catenin was clearly localized in the cellular membrane and cytosol region, suggesting that the Wnt/β-catenin signaling pathway was seldom aberrant in our samples. Since original tumors were poorly differentiated, the expression of *CDX2* was relatively low in all tumor samples and their derivatives. The expression of *SMAD4* was depleted in all tumor tissues, with very weak staining observed in organoid C-TO, E-TO, and F-TO samples. A few organoids exhibited different staining intensities of CDX2 and SMAD4 from their corresponding tumors. Spatial heterogeneity within a single tumor mass may account for the differences. Although the tissue was thoroughly disseminated, the tumor mass that was subjected to in vitro culture spatially varied from the tissue section used for IHC. With respect to the intensities of expression, the previous studies of IHC of CRC organoids have reported that CDX2 expression was intensified in passaged organoids compared to the original tumors^5^.

We also successfully generated patient-derived organoid xenograft (PDOX) models. This transplantation approach allows primary tumor formation under mouse skin, thus providing an applicable microenvironment for PDOs. It can retain homogeneity regarding histological features, tumor-specific protein, genetic and transcriptome characterization, and drug response of the original patient tissues and organoids^6^. H&E staining and IHC of a few characteristic biomarkers validated that PDOX models were highly analogous to matched donor tumors and organoids in the secondary architecture of malignant glands (Supplementary Fig. 1b).

Overall, these data demonstrate that we have successfully established PDOs, PDCs, and PDOCs, with PDOs retaining histological characters of the corresponding six different regions of one synchronous intestinal tumor patient.

### Multi-regional organoids and cell lines retain genomic features with moderate genetic heterogeneity of human synchronous intestinal cancer

**Fig. 2:**
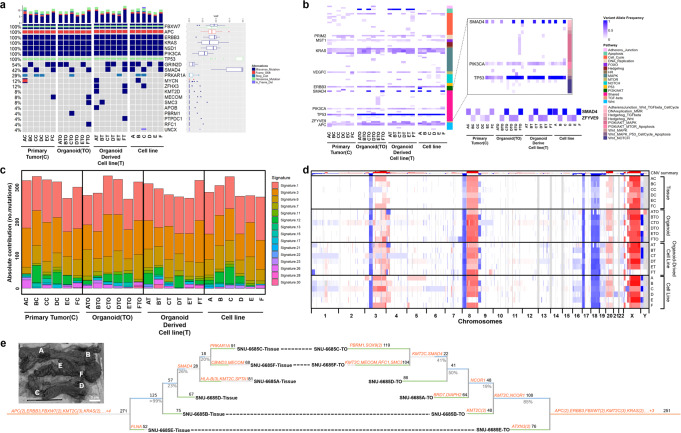
Sub-regional analysis of genomic characteristics of PDOs and PDCs revealed evolutionary trajectories of SIC. See also Supplementary Figs. 2 and 3**. a** Genomic profiles of original tumors and their derivatives of the patient, SNU-6685. Multiple somatic mutations including point mutations in putative tumor driver genes were identified. Variant allele frequencies (VAFs) of somatic mutations are indicated with a box plot on the right side of the heatmap. Each specific type of alteration is marked with representative colors. **b** Heterogeneous distributions of VAFs in somatic mutations involved in multiple signaling pathways. Derivatives regardless of the different culture methods preserved VAFs of corresponding tumors in the majority of cell signaling pathways (*n* = 11). Within a single patient, genes regulating TGF-β signaling pathways such as *SMAD4* and *ZFYVE9* exhibited subregional clone-specific VAFs. **c** Total mutational load and mutational signatures of derivatives and paired primary tumors. The different colors represent different signatures (*n* = 30). **d** Genome-wide copy number variations (CNVs) of synchronous tumor tissues and their derivatives (red, gains; blue, losses; white, diploid). CNV patterns were analogous regardless of different culture methods as well as the multi-focal tumor sites. **e** Phylogenetic trees illustrating the evolutionary trajectory of SIC tissues and corresponding PDOs. Based on multiregional WES profiles, Treeomics classified somatic mutations as the trunk (orange), shared (blue), and individual (green). Dotted lines indicate the genetic hierarchical position of each site was mostly analogous between tissues and PDOs. Numbers on top of each branch indicate the number of acquired variants. Genes that are listed in the CGC are shown in orange. Numbers on the bottom of each branch indicate estimated support values. The actual photograph of the tumor shows the marked sites from which each subclonal derivative was obtained (A–F). Scale bars = 5 cm.

We analyzed genomic profiles including somatic mutations and copy number variations (CNVs) of both original tumors and matched derivatives using whole-exome sequencing (WES). The detailed yield of sequencing experiments is summarized in Supplementary Data 2a. Adjacent normal mucosa was used as a control to discrete somatic mutations and provide baseline CNVs in each sample. Of the total somatic mutations (*n* = 11,601) including SNVs and indels, the percentage of overlapping mutations between original tumors and derived models ranged 32.73–46.38%, 42.74–57.45%, and 52.18–63.13% in organoids, organoid-derived cell lines and cell lines, respectively (Supplementary Data 2b). For analyzing representative genetic mutations, we narrowed genes of interest down to previously published pan-cancer driver genes^7^ (*n* = 299). The mutational landscape of PDOs, PDCs, and PDOCs indicated that all derivatives retained >75% of variants in each corresponding tumor. Besides, the overlap of mutations in the coding region between tumor regions revealed extensive intertumoral heterogeneity. Of the overall somatic mutations (*n* = 982) detected in tumor samples, 116 mutations (12%) were commonly found in all tumor regions, and only 5–20 mutations (0.51–2%) were shared between tumors. The calculation using PDO data was higher compared to tumor tissues. A total of 1032 mutations were detected in PDO samples, 108 mutations (10%) were concordantly found in all PDO regions, and 6–26 (0.58–2.5%) mutations were overlapped between PDOs. Our result was accordant with previous studies of SIC that reported intersection of mutations between tumors was consistently low^4^. Prevalent genetic alterations in CRC such as inactivating mutations in *FBXW7* (p.Try545Cys and p.Arg479*), *APC (*p.Thr621fs and p.Ser1415fs), and *TP53* (p.Arg306*) as well as activating mutations in *KRAS* (p.Gly12Phe) and *PIK3CA* (p.Glu545Gly) were detected in all tumors with variant allele frequencies (VAFs) of ~0.5 except for *TP53* [variant allele frequency (VAF) = ~1.0]. Although all multifocal sites harbored pathogenic *APC* mutations, this patient had no highly penetrant causative mutations of familial adenomatous polyposis (FAP), Lynch syndrome, and other familial conditions, including *AXIN2*, *POLD*, *MYH*, other MMR-related genes^8^. In contrast, sub-lineage-specific mutations were found with relatively lower VAFs (~0.3) (Fig. 2a, Supplementary Data 2c, d). This suggests that prevalent driver mutations might have initiated tumorigenesis by altering cell proliferation, differentiation, and apoptosis as genetic background, but larger portions of site-specific gene alterations account for independent tumor development and in turn caused high inter-tumor heterogeneity within a single patient^9^ (Fig. 2a, Supplementary Data 2c, d). Notably, one specific *SMAD4* alteration (K436M) was site-specifically detected in A, C, and F sites with VAFs of ~1.0. *SMAD4* is an essential tumor suppressor gene that can mediate transforming growth factor-β superfamily signaling^10^. Its allelic imbalance in chromosome 18q21 has been associated with poor prognosis in CRC patients^11^. This implies that another ancestry progenitor might have been generated from the aberrant *SMAD4* and then proceeded to each site-specific malignancy which might have developed a genetic heterogeneity. We then compared VAFs of somatic mutations involved in various cellular processes and cell signal transductions between original tissues and their derivatives. Although we used low-passaged derivatives (p2–4) in order to minimize the effect of repetitive subculture, mutations detected from tumor tissues had relatively low VAFs compared to their derivatives partially due to lower cellularity of the resected tumor and the selection process caused by in vitro subcultures. Especially, the majority of known tumor driver mutations had lower VAFs in tumor tissues compared to their derivatives (Fig. 2b; Supplementary Data 2d, e).

We then compared the mutational signatures of each site-specific tumor and its derivatives. Overall, mutational concordance of multi-site cultures within coding regions corresponded closely with matched tumor specimens, showing that the predominant point mutation type remained unchanged throughout the tumor progression (Supplementary Fig. 2a, b). High contents of C to T transition, which is featured in mutational signature 1 and common in sporadic colorectal cancer^12,13^, were consistently predominant across different tumor sites as well as sample types. Mutational signature 3 which is associated with BRCA1/2 mutations and signature 6 which is related to defective DNA mismatch repair (MMR) genes were also identified. Those signatures were unexpected considering that this SIC case was microsatellite stable and had no germinant/somatic mutations in MMR nor BRCA1/2 genes (Fig. 2a, Supplementary Data 2d). The algorithm estimating the absolute contribution of substitution patterns was to fit 96 types of substitution into previously constructed mutational signature (Supplementary Data 2f). Therefore, a single substitution can be assigned to multiple signatures, which is likely to be interpreted as other signatures. We further analyzed the mutational signature of tumor tissues and corresponding organoids constructed by the relative contribution of substitution patterns. This revealed the overall contributing shape largely resembled the mutational signature 1, which is characterized by high contents of C to T transition (Supplementary Fig. 2c, d).

Nearly 85% of CRC cases are complicated by chromosomal instability^14^. Especially, the losses in chromosome 8 are associated with lymph node and distant metastases in addition to disease susceptibility^15^. Our exome-wide copy number variations (CNV) analysis revealed that gain at chromosomes 3, 8, and X and loss at chromosome 18 were uniformly presented regardless of culture type (Fig. 2d). Our samples exhibited distinctive abnormalities of chromosome 3 compared to the TCGA-COAD cohort (Supplementary Fig. 3a). Genomic instability on chromosome 3 has been associated with inherited cancer syndromes mainly due to *MLH1* gene^16^. Nevertheless, manual inspection of the aberrant region in chromosome 3 revealed that the copy number of *MLH1* was rarely affected. Instead, multiple genes involving epigenetic regulations such as histone methyltransferase (*SETD2*), SWI/SNF complex (*PBRM1*), and histone modifier (*BAP1*) showed copy number loss (Supplementary Fig. 3b). Several studies reported that the loss of such genes caused uncontrolled cell proliferation and decreased genome integrity^17,18^. This suggests that post-translational modification to histone proteins accounts for further tumor progression by altering chromatin structures or recruiting histone modifiers.

To consolidate genomic features determining evolutionary factors contributing to intra-patient heterogeneity as well as a clonal association within these synchronous tumors, we conducted a phylogenetic analysis of multi-site sequencing data using Treeomics algorithm^19^ (Fig. 2e). On the basis of multi-site mutational profiles including their VAFs, the algorithm classified somatic mutations as the universe (trunk), more than two sites (shared), and a specific site (individual). This analysis facilitated us to follow evolutionary trajectories of multi-site tumors and confirmed that our PDO models recapitulated evolutionary dynamics within the original tumor. In order to access the candidate mutations that initiate tumorigenesis along the bowel tract, we narrowed the genes of interest by referring to the Cancer Gene Census list^20^ with minimum VAFs of 0.2. We identified that alterations in Vogelgram^21^ genes such as *APC* (c.1861dupA/p.Thr621fs, c.4245delT/p.Ser1415fs), *FBXW7* (c.1634A>G/p.Tyr545Cys, c.1435C>T/p.Arg479*), *KRAS* (c.35G>T/p.Gly12Val, c.34G>T/p.Gly12Cys) and PIK3CA (c.1634A>G/p.Glu545Gly) and TP53 (c.916C>T/p.Arg306*) initiated the development of tumor as these mutations were found in the trunk of the phylogenetic tree. Tumor site E (first small bowel) was adjacent to these ancestry mutations (*n* = 272) and other 52 site-specific mutations contributed to tumor progression. Likewise, tumor sites B (transverse colon) and D (rectum) also progressed with 75 and 67 site-specific mutations respectively, accounting for further intertumoral heterogeneity. Afterward, one more ancestry clone was formed centering around *SMAD4* (c.1307A>T/p.Lys436Met) mutations with VAFs of ~0.5 at sites A (ascending colon), C (sigmoid colon), and F (small bowel). Tumor A has accumulated several driver mutations such as KMT2C (c.5564C>T/p.Pro1855Leu) with VAFs of ~0.5 indicating one major branch was formed by epigenetic factors. The tumor C further progressed with stop loss mutation of *PRKAR1A* (c.1012T>G/p.Ter338Gluext*) with VAFs of ~0.18. The tumor F developed with MECOM (c.191A>G/p.Tyr64Cys) with VAFs of ~0.2. This suggests that tumor sites A, C, and F were actively evolving at the time of surgical resection reflected by the relatively high tumor mutation burden. By following evolutionary trajectories of this SIC case, we confirmed that site-specific mutations consisted of intertumoral heterogeneity, yet multifocal tumors were clonally related with multiple shared mutations such as SMAD4 (c.1307 A>T/p.Lys436Met). An analogous pattern was observed in the phylogenetic tree of PDOs. Both trees indicated that patterns of tumor progression and distribution of subregional mutations were seldom in an organ-specific manner. Instead, the mutational distance between site C (sigmoid colon) and site F (small bowel) was adjacent to each other in this patient (Fig. 2e). With respect to mutational accumulations, our result highlighted that the evolutionary distance and clonal relationships among six tumor sites along the bowel tract was coincided neither with the anatomical distance nor with the organ-specific microenvironment.

### Gene expression analysis demonstrates transcriptomic heterogeneity within multi-sampling intestinal tumor

**Fig. 3:**
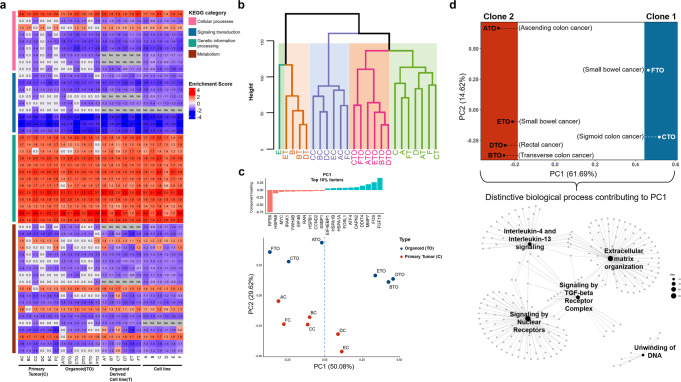
Transcriptomic analysis demonstrated distinct mRNA expressional patterns according to different clonality. Related to Supplementary Fig. 4 and Supplementary Data 3**. a** Gene Set Enrichment Assay (GSEA) analysis using normalized transcripts of all genes (*n* = 35,993) of tumor tissues and corresponding derivatives compared to the normal tissue. The KEGG signaling pathway pathways are categorized and indicated with representative colors. A total of overlapped 50 pathways that were enriched in both tumors and PDOs were selected to identify the differentially expressed pathway between samples (red, upregulated; blue, downregulated). **b** Sample to sample distances of mRNA expressions of all genes (*n* = 35,993) among tumor tissues (*n* = 6) and corresponding derivatives (*n* = 18) based on hierarchical clustering. Each cluster is marked with representative colors. The intact cluster of organoids was adjacent to the original tumors in Euclidean distance. **c** Principle component analysis (PCA) using values of normalized transcripts indicates tumor site D and E from the rest with principle component 1 (PC1) whereas principle component 2 (PC2) divided PDOs from tissue. Top 10% contributing variables in PC1 are indicated on the top of the PCA plot. **d** Gene Ontology of the top 5% genes contributing to the distinction of C- and F-TO from the rest (PC1) was performed with *P*-value < 0.05 as a cutoff. Among the selected 21 pathways in gene sets of the Reactome database, five enriched biological processes (*p*-value < 2.16E−05) were indicated in cnetplot.

We also conducted a genome-wide transcriptomic analysis of a total of 25 samples, including mRNA expression data from original tumor tissues with normal mucosa and corresponding tumor derivatives. We first compared enriched pathways in these multifocal SICs and derivatives compared to normal mucosa by performing a gene set enrichment assay (GSEA) analysis based on read counts of all genes (*n* = 35,993). False discovery rate (FDR) < 0.25 of normalized enrichment score (NES) was used as a cutoff to select 50 tumorigenesis-related KEGG signaling pathways. The heatmap of NES indicated that PDOs, as well as PDCs overall, recapitulate the pathway enrichment of corresponding tissues. Detailed information on analyzed pathways is summarized in Supplementary Data 3a. All gene sets related to genetic information processing were enriched in both tissues and derivatives compared to normal control. We observed that gene sets of “Cell_Cycle” and “P53_signaling_pathway” were highly enriched whereas “Apoptosis” was relatively depleted compared to normal mucosa which possibly resulted in cancer progression. Moreover, gene sets involved in cell signal transduction were downregulated in all samples, suggesting that these aberrant signaling pathways were involved in the progression of synchronous tumors within the bowel tract^22^ (Fig. 3a; Supplementary Data 3a). Our data also demonstrated that PDOs better-retained pathway enrichments of corresponding tumors especially in “Cellular pathway” and “Metabolism” categories compared to PDCs.

Then, we additionally estimated the transcriptomic distance between multifocal sites as well as their derivatives by using normalized transcript expressions (transcripts per million, TPM) of 35,993 genes. Hierarchical clustering analysis demonstrated that expressional patterns mainly depended on diverse culture methods rather than on site-specific transcriptomic features, considering that cell lines and organoids mainly consisted of epithelial cells whereas tumor tissues were infiltrated with mesenchyme, blood vessels, and immune cells aside from epithelial cells^23^. The intact cluster of organoids was adjacent to the original tumors in Euclidean distance, validating that PDOs could better represent their matched primary tumors than other culture types in spite of the inevitable variances from in vitro cultivation. With this analysis, we included data exclusively from the tissue and PDOs to further analyze transcriptomic landscape of multifocal synchronous tumors (Fig. 3b).

We examined the extent of transcriptional heterogeneity between multi-focal tumors using principal component analysis (PCA). We used the normalized value of transcripts (*n* = 35,993) in the original tumors (AC-FC), which are log2 transformed values of tumor TPM/normal mucosa TPM. With respect to transcriptomic patterns of synchronous tumors, 85.12% of transcriptomes accounted for scattered patterns of principle component 1 (PC1) and principle component (PC2) after removing the lower 10% of variables based on variance (Supplementary Fig. 4a). This suggested that each multifocal tumor distinctively regulates gene expressions in synchronous intestinal cancer. We further investigated that the derived models also retained expression heterogeneity identified in synchronous tumors. To discover the overlaps of transcriptomes that affect intertumoral heterogeneity between tumors, we collected the top 10% of genes that contribute to PC1 of the original tumors and their derivatives including PDOs, PDOCs, and PDCs, respectively (Supplementary Fig. 4a, b, Supplementary Data 3b). Although transcriptomes of cancer cell lines and organoids are likely affected by varying composition of culture media and cell density^24^, contributing components (genes) that accounted for heterogeneous patterns of the original tumor were intersected more than 57% of their derivatives.

We also have estimated the consensus molecular subtypes (CMS) of each tumor and its derivatives using R package, CMScaller^25^. We used raw read counts of six synchronous tumors as an input with ‘RNA-seq = True’ setting. The analysis indicated that tumor tissue AC had CMS type 2 and the rest had CMS type 4 (Supplementary Fig 4c, Supplementary Data 3c). CMS subtyping using mRNA expressions is largely based on representative molecular pathways such as DNA repair, cell cycle, and EMT (Supplementary Fig 4d, Supplementary Data 3d). This revealed that the transcriptomic heterogeneity observed in hierarchical clustering analysis and principle component analysis was independent to such molecular pathways, suggesting that the expressions of less characterized genes were determinant to the transcriptomic heterogeneity of this SIC case. Figure 3a reflected these results as the enrichment score of most signaling pathways was analogous to each other.

We then investigated specific genes contributing to the separation of multifocal tumors. To examine those genes that influenced differential expression among multi-site clones, we narrowed genes of interest to gene sets consisting of 11 cancer-related signaling pathways from the Kyoto Encyclopedia of Genes and Genomes (KEGG) database (Supplementary Data 3e). PC2 clearly separated PDOs from tissue samples as expected based on the results of the prior hierarchical clustering analysis. In parallel with previous findings from the phylogenetic analysis (Fig. 2e), tumor sites CC (descending colon) and FC (small intestine) were closely correlated regardless of the organ specificity based on PC1 which more than half of the genes contributed (Fig. 3c; Supplementary Data 3f). This reconfirmed the strong molecular association between sites C and F. RPS6 gene encodes a component of the 40S ribosomal subunit associated with the mTOR pathway^26^. It was the most influential loading component that clustered organoids A-, C-, and F-TO and their matched tissues among the top 10% loading components. FGF19 mRNA expression was highly upregulated and correlated with tumor DC and EC and organoids B-, D- and E-TO (Supplementary Data 3f).

Further analysis proceeded from the principal components which spatially separated PDOs. The top 5% contributing factors (*n* = 3598) within the loading components of PC1, which showed both negative and positive correlations, were mapped with a genome-wide annotation database for humans using org.Hs.eg.db (v3.10.0) R package. The detailed analysis process can be found in the “Methods” section. Genes related to “Extracellular Matrix Organization”, “Signaling by Nuclear Receptors”, “Signaling by TGF-beta Receptor Complex” and “Interleukin-4 and Interleukin-13 signaling” were differentially expressed between C-TO, F-TO and the rest of PDOs (Fig. 3d; Supplementary Data 3g). Overall, these data validated that the expressional patterns of canonical pathways in multifocal SICs were highly altered with respect to the normal tissue and replicated the result from mutational analysis. Among the derived models, PDOs retained the patterns of multi-regional transcriptomic heterogeneity as well as characteristics of clonal association identified in synchronous tumors.

### Heterogeneous epigenetic changes affect DNA repair system, leading to different tumor development patterns

**Fig. 4:**
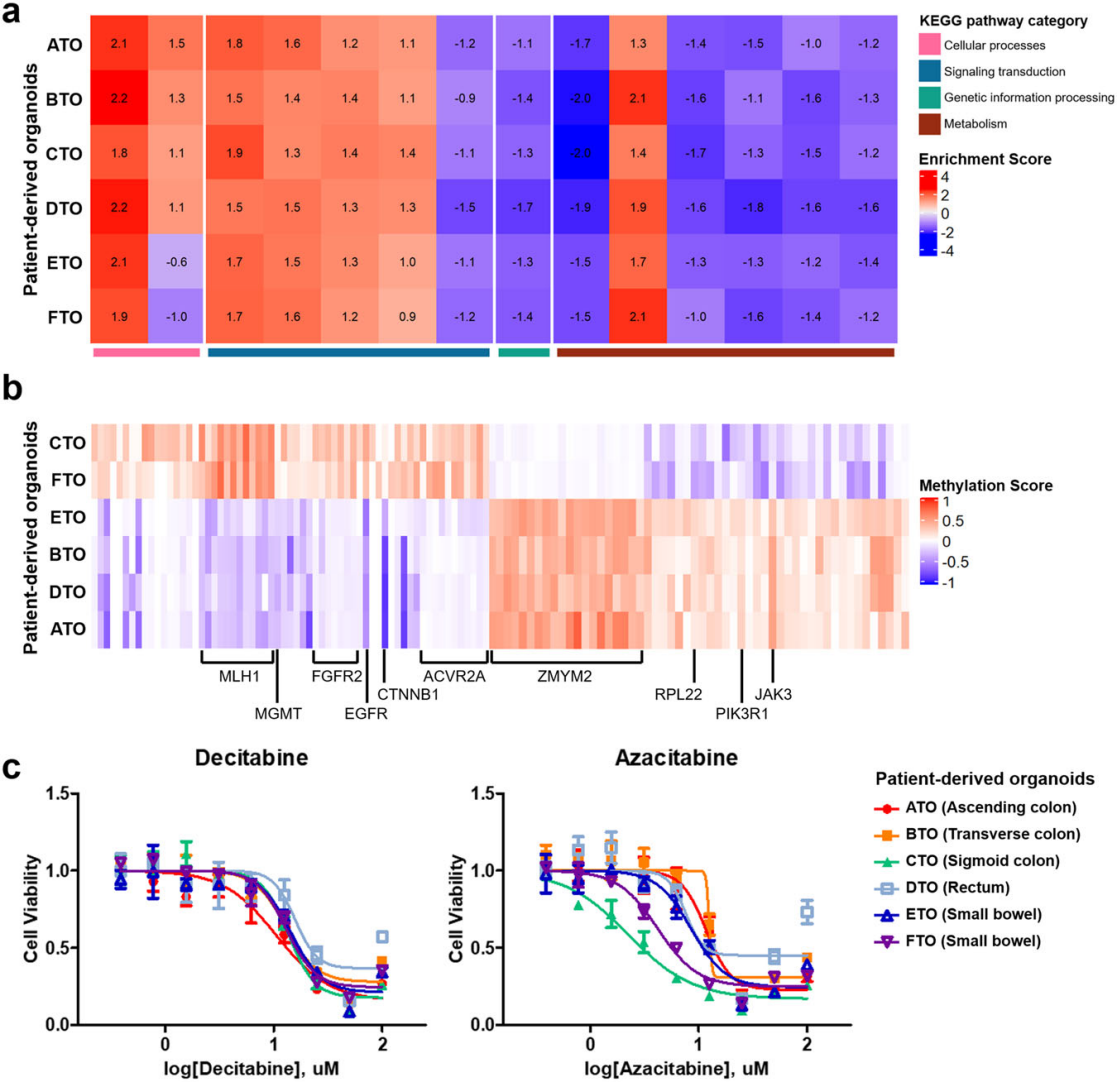
Heterogeneous epigenetic changes affect DNA repair system, leading to heterogeneous tumor development patterns. See also Supplementary Fig. 5. See also Supplementary Data 4**. a** GSEA analysis using normalized promoter methylation score of PDOs. The KEGG signaling pathway pathways are categorized and indicated with representative colors. Tumor-related pathways (*n* = 14) were significantly dysregulated compared to the normal mucosa. **b** Differentially methylated genes separating C and F-TO from the rest (standard deviation < 0.1). Hypermethylated promoter regions (*n* = 63) and hypomethylated promoter sites (*n* = 54) were identified (red, hypermethylated; blue, hypomethylated). **c** The sigmoid curves of PDOs treated with two different types of methyltransferase inhibitors. Each PDO is marked with representative colors. The error bars represent the standard deviations (*n* = 3).

Previous studies about the synchronous intestinal tumor seldom investigated the epigenetic profiles of multifocal tumors. Only a few studies on epigenetic profiling of synchronous colorectal neoplasia have reported that promoter methylation patterns between synchronous tumors were highly analogous^27,28^. Organoid technology has been widely used to retain the region-specific DNA methylation patterns and regional gene expressions. Especially, intestinal organoids from the human small intestine and colon have global methylation patterns and epigenetic ages similar to the primary crypt cells from which they were derived^29^. We evaluated the DNA methylation profiles across six different tumor sites using established PDOs. We first compared the enrichment scores of tumor-related pathways among multifocal PDOs using a single-sample gene set enrichment analysis (ssGSEA) of methylation status compared to normal mucosa, which revealed that 14 tumor-related pathways were significantly dysregulated. Although pathway enrichment scores across six different PDOs were mostly analogous to each other, we demonstrated that aberrations with cell proliferation, communication, and genetic process pathways accounted for the onset of this SIC case (Fig. 4a; Supplementary Data 4a). “Focal_Adhesion” pathway exhibited an organ-specific enrichment score, in which it was specifically depleted in PDOs derived from the small bowel (E-TO and F-TO) compared to the rest (Supplementary Data 4a).

We further inspected heterogeneously distributed methylation enrichment scores among PDOs. We calculated the Euclidean distance of methylation pattern of 14,434 methylated CpG sites in promoter regions of pre-selected tumor-related genes (*n* = 299) between various tumor sites-derived organoids (Supplementary Fig. 5), which revealed that C-TO (sigmoid colon cancer) and F-TO (small bowel cancer) were tightly grouped together, and the rest shaped another cluster. In order to further investigate which regions accounted for the distinct epigenetic patterns of C-TO and F-TO, we selected the specific regions in which the standard deviation (SD) of methylation scores (m) between C and F organoids was <0.1. This enables us to assign the 63 promoter sites where m of C and F-TO ≥ 0 as CF-specific hypermethylated regions and the 54 promoter sites where m of C and F-TO < 0 as CF-specific hypo-methylated regions. The heatmap in Fig. 4b summarized this result using the color variance ranging from −1 (hypo) to 1 (hyper). Genes that encode DNA damage repair proteins such as *MLH1* and *MGMT* were hyper-methylated in C and F-TO compared to the rest as indicated in Fig. 4b. This might account for the relatively high mutational burden in C and F-TO (Fig. 2c). Besides, the promoter region of multiple genes involved in the regulation of cell cycle, proliferation, and migration such as membrane receptor in TGF-β signaling (*ACVR2A*), FGF signaling (*FGFR2*), and WNT signaling (*CTNNB1*) pathways was specifically hyper-methylated in C and F-TO. In contrast, the rest of PDOs were hyper-methylated in regions encoding chromatin regulator (*ZMYM2*) and cytoplasmic ribosomal protein (*RPL22*) (Fig. 4b; Supplementary Data 4b). Such heterogeneous methylation profiles suggested that post-translational regulation was considerably involved in further tumor progression and diverse clonal expansions of SICs.

Next, we examined the responses of PDOs to two different methyltransferase inhibitors, azacitidine and decitabine, in order to demonstrate that a heterogeneous pattern of DNA methylation affects the sensitivities of clinically applicable drugs. The EC50 values indicated that azacitidine responded more than three times better than decitabine in C and F organoids, which was in parallel with genomic, transcriptomic, and epi-genomic patterns. Both azacitidine and decitabine inhibit cell growth by inhibiting methylatransferase, yet azacitidine is capable of interfering into RNA to a larger extent than into DNA whereas decitabine is only incorporated into DNA. Considering the better responses of azacitidine than decitabine in organoid C- and F-TO (Fig. 4c, Supplementary Data 4c), we deduced that specific RNA hyper-methylation in C and F organoids accounts for the particularly effective response of azacitidine. Overall, these data validate that considerable epi-genomic heterogeneity exists within SICs, which reflects different regulations of multiple pathways including cell growth, signaling transduction, DNA replication, transcription, and translation in accordance with mRNA expression as well as different responses to methyltransferase inhibitors among PDOs.

### Heterogeneous drug responses of synchronous intestinal tumors depend on site-specific tumor heterogeneity

We have prepared a 25-drug screening library to measure heterogeneous drug responses of synchronous colorectal tumors. We have mainly referred to National Cancer Institute(NCI)-approved drugs (https://www.cancer.gov/about-cancer/treatment/drugs/colorectal#1) for colorectal cancers (CRCs) for constructing the drug screen library. We also have referred to the actual clinical history of chemotherapy. After surgery, the patient received 4 cycles of FOLFOX with Bevacizumab as well as 4 cycles of FOLFIRI with Bevacizumab. According to this clinical history, we included fluorouracil, oxaliplatin, and irinotecan in the screening library. Considering the genetic and epi-genetic heterogeneity of SIC, we also included several targeted drugs such as afatinib, regorafenib, and trametinib as well as traditional chemotherapies for CRCs such as fluorouracil, irinotecan, and capecitabine in order to compare the variable responses of multisite tumors in accordance with their molecular features. Specifically, we involved several drugs that regulate DNA/RNA methylation such as azacitidine and decitabine because of highly different epi-genomic landscapes. All PDOs, PDCs, and PDOCs were screened in triplicate, producing > 1200 measurements of drug interactions. We used the same PDCs and PDOs that were subjected to molecular sequencings in order to optimally reflect the molecular features of preclinical models to drug responses. We also used a complete Human Intestinal Stem Cell (HISC) medium for both PDOs and PDCs through the entire procedure for drug screening to minimize the possible variables from using different culture mediums.

The Elbow method indicated that there were four optimal clusters in both drugs and derivatives (Supplementary Fig. 6a, b). *K*-means clustering of AUCs according to the number of clusters from the elbow method identified three major sub-groups among screened compounds. Distances between clusters are recomputed at each stage by the Lance–Williams dissimilarity update formula according to the *k*-means method. Two of the most similar clusters are joined at each stage until there is a single cluster. Two-way analysis of variance (ANOVA) was applied to demonstrate the first drug group including fluorouracil, buparlisib, trametinib, apitolisib and belinostat displayed good responses to all derivatives regardless of varying culture methods (Supplementary Data 5b). The second group exhibited heterogeneous sensitivities across multiple PDOs. Notably, drugs targeting epigenetic regulators such as histone deacetylate inhibitor SAHA, decitabine, and azacitidine had highly variable AUC values, reflecting heterogeneous epigenetic profiles among multiple subregional clones. The third and fourth groups showed moderate to high insensitivities across all PDOs regardless of the resected site (Supplementary Fig. 6c, Supplementary Data 5a).

**Fig. 5:**
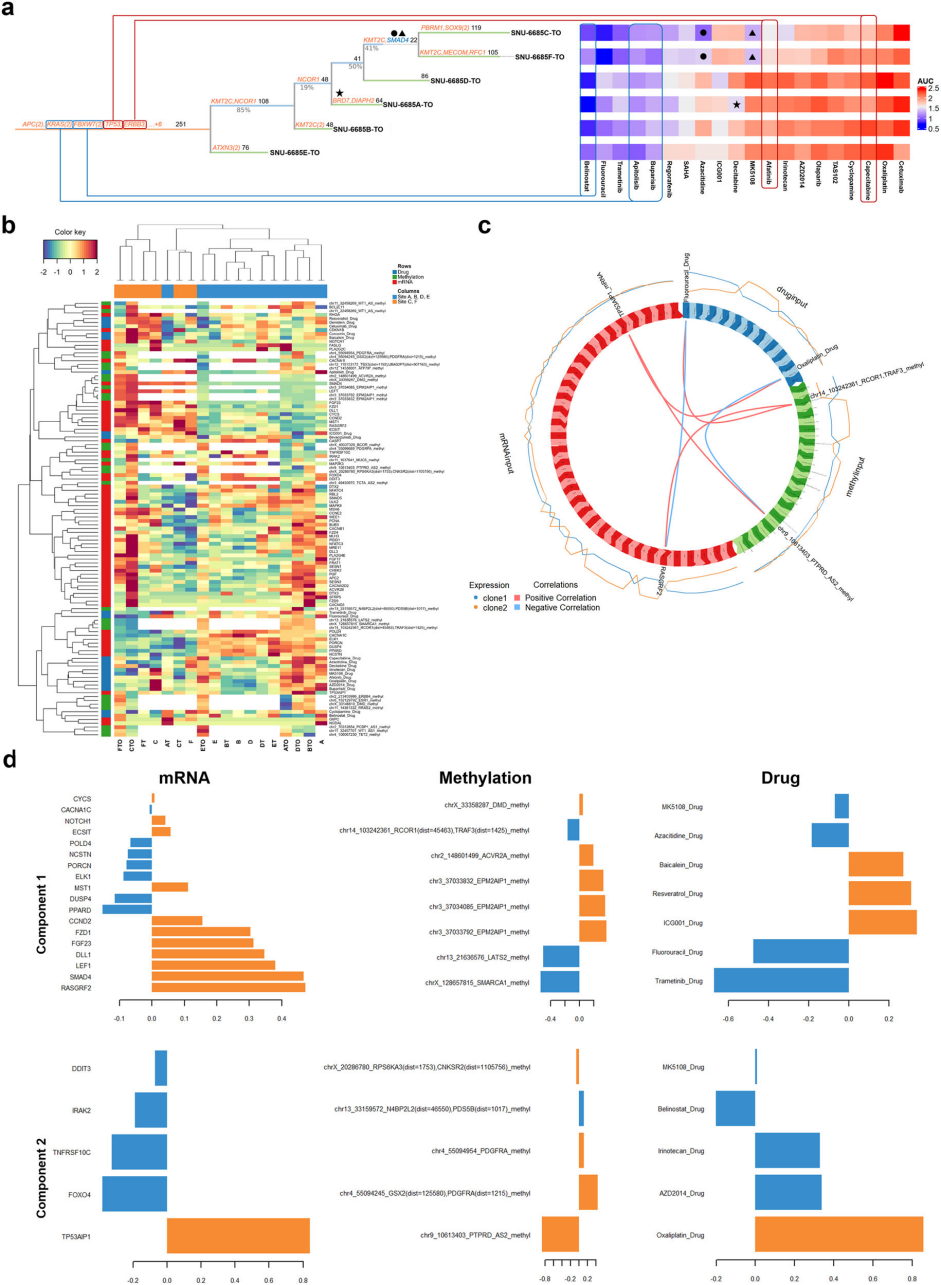
Heterogeneous drug responses of synchronous colorectal tumors depend on site-specific tumor heterogeneity. See also Supplementary Fig. 6**. a** Multifocal organoids displayed heterogeneous drug responses in accordance with various branch mutations. Universal mutations detected in all multifocal organoids accounted for similar responses among subregional clones (marked with red lines, poor response; blue lines, good response). Branch mutations that were partially observed among subregional clones were related to heterogeneous drug responses (marked with plane figures). Previously known driver genes that are listed in the CGC were highlighted in orange and those related to drug responses were indicated in blue. **b** According to the clonal association identified in drug responses, tumor sites C, F, and the rest of the derived models were designated as clone 1(orange) and clone 2(blue) respectively. Transcripts (*n* = 759), methylated promoter sites (*n* = 328), and AUC values of 25 chemical compounds separating subregional clones derived from sites C and F from the rest were integrated. Multivariate values including mRNA expression, methylation, and drug response were normalized by Weight Standardization and ranged from −2(blue) to 2(red). Only the top 10% variables (*n* = 114) were selected to confirm the significant correlation between multiple variants. Parameters that highly separate sites A, B, D, and E from sites C and F are clustered on the left side. **c** The positive and negative correlation between parameters is displayed with circos plot. (red, positive correlation; blue, negative correlation). Two-component connecting 3 variables were selected by the cutoff of correlation coefficient values over 0.8. In accordance with the drug responses, derivatives from sites A, B, D, and E were designated as clone 1. The rest was labeled as clone 2. **d** Two components separating sites A, B, D, and E from sites C and F. Their parameters accounted for the different clonality. In accordance with the drug responses, derivatives from sites A, B, D, and E were designated as clone 1. The rest was labeled as clone 2.

We then manually inspected the association between specific mutations and drug responses by referring to gene-drug databases such as the Drug Gene Interaction Database (DGIdb)^30^. Previously known driver genes that are listed in the Cancer Gene Census (CGC) were highlighted in orange and those related to drug responses were indicated in blue. Mutations that were detected in the trunk of the phylogenetic tree were associated with identical sensitivities among multifocal organoids (marked with red lines, poor response; blue lines, good response). Heterogeneous distribution of specific mutations among subregional clones accounted for divergent drug responses (marked with plane figures). For instance, two-way ANOVA indicated that all subclones showed a good response to apitolisib in accordance with the KRAS mutation (c.34_35GG > TT) (Supplementary Data 5b). Also, independent SMAD4 mutation (c.1307 A > T/p.Lys436Met) that accounted for the separation of C-TO and F-TO from the rest of PDOs on the phylogenetic tree was associated with sensitivity of multiple drugs including azacitibine and MK-5108 (Supplementary Data 5c). AUC values for both drugs were significantly lower in these two clones than in other subregional clones. From this perspective, the clonal relationship between C- and F-TO, which were independently distinct from the trunk in the phylogenetic tree by independent mutations including SMAD4, coincided in the drug responses between PDOs. In a similar context, only A-TO clone had BRD7 mutations which are known to be related to decitabine sensitivity (Fig. 5a).

We further validated various associations connecting mRNA expression levels, methylation scores, and drug responses. We pre-selected mRNA expression levels of 759 genes associated with the tumorigenesis (Consensus Geneset) and 328 methylation sites, which were clustered into two subregional groups as clone 1 (*n* = 6, derivatives from tumor sites C and F) and clone 2 (*n* = 12, derivatives from the rest of tumor sites). These preprocessed expression values were used for the input of multi-omics analysis. We implanted latent components (linear combinations of variables) are being constructed in order to maximize the sum of covariance between clone 1 and clone 2 in the datasets. Among various factors, elements that separated clones C and F from the rest (*n* = 114) were selected and clustered across the colored heatmap as a multi-omics signature (Fig. 5b). For hierarchical cluster analysis, the two clusters that had the most analogous patterns were combined at each process until a single cluster left. Each variable was allocated to a unique cluster, which iterative algorithm proceeded. Correlation scores for each element were then calculated. Among the statistically significant elements (*p* < 0.05), the correlation circos plot of the multi-omics signature represented two components at a confidence level of 0.95 for the parameter. Each variable coordinate is defined as the correlation between the original variable value and each component. A correlation circle plot enables us to visualize the correlation between variables (Fig. 5c). The first component consisted of TP53AIP1 (mRNA expression), chr9_10613403_PTPRD_AS2 (methylation score), and oxaliplatin (drug response). The sensitivity of oxaliplatin was positively correlated with mRNA expression of TP53AIP1 but negatively associated with the degree of methylation of PTPRD. The mRNA level of TP53AIP1 increased methylation of promoter regions in PTPRD gene, which in turn possibly diminished the protein level PTPRD, thereby sensitizing tumor cells to oxaliplatin. The second component consisted of RASGRF2 (mRNA expression), chr14_103242361_RCOR1, TRAF3 (methylation score), and fluorouracil (drug response). Drug (fluorouracil) response was negatively correlated with mRNA expression of RASGRF2 but positively associated with methylation scores of RCOR1 and TRAF3. The mRNA level of RASGRF2 positively contributed to the degree of methylation at RCOR1 and TRAF3 promoter sites, thereby decreasing their protein expression and eventually leading to a good response to fluorouracil (Fig. 5c). Using a correlation cut-off of 0.8, weights were assigned to each element in accordance with clonal separation (Fig. 5d). The coefficients of the loading vectors contributing to each component are shown in the bar chart. Their absolute values indicate the extent of influence of each variable to determine its component. Overall, we identified the associations between methylation and mRNA expression on pathways with biological significance and synchronous tumor-derived models retained the traditional understanding of the linkage with methylated CpG sites of gene promoter region and reduction of gene expression^31,32^. These two sets of inter-correlations connecting three different molecular levels of omics data also reemphasized the effect of integrative actionable targets on certain drug responses in the previous studies^33^. Since the treatment of clonally variable synchronous neoplasia requires clone-specific inspection of variable molecular layers in clinical practice^3^, our multi-omics platform enabled a more precise distinction of clonal difference in synchronous tumors, which provides superior clinical application.

## Discussion

Patients whose primary cancer involves synchronous tumors generally have poorer prognoses than patients with metachronous tumors mainly due to the unexpected resistance to certain chemotherapies caused by variable clonality in multi-regional tumor masses^34^. In the surgical findings, 4 of 6 lesions had not invaded the serosal side of the colon and had only invaded the subserosal side of the colon. When the tumors from peritoneal seeding invade the other site of the colon, the invasion occurs from the serosal side of the colon (the most outer side) to the mucosal side (the most inner side). On the other hand, primary colon cancer starts from the mucosal side of the colon and invades the serosal side of the colon. Hence, the tumors may be synchronous lesions rather than an outer invasion of seeding tumors. However, there is no molecular consensus standard with which to classify multifocal colorectal cancer as a synchronous primary tumor or intraluminal seeding^35–37^. According to the recent findings^3^, the intraluminal spread is considered multifocal colorectal cancers, which are caused by inherited predisposition and development of secondary tumors that are not related to the primary tumor. The status of microsatellite instability is also to distinct multifocal colorectal cancer. The enrolled patient in this study was microsatellite stable and had no inherited predisposition, which is considered sporadic synchronous cancer. The majority of previous SIC research studies were focused on clinicopathological characteristics of the SIC. Comprehensive molecular analysis with the perspective of heterogeneous drug responses of all synchronous tumors within a single patient has not been reported yet.

In this study, we successfully established in vitro models of PDOs and PDCs directly after surgical resection. We demonstrated that genetic features of original SIC tumors were well retained in both PDOs and PDCs. These derivatives, especially PDOs, captured the inter-tumoral heterogeneity within a single SIC patient, which was reflected by various responses to targeted therapies. Ancestry mutations detected in SNU-6685 patients were mostly known driver genes in CRC^38^, suggesting that genes accounting for the initiation of SIC are analogous to Vogelgram genes. The mutational status of *KRAS* was noteworthy as distinct *KRAS* mutations in codon 12 (c.34_35delGGinsTT) were observed in all synchronous tumors. We confirmed that the two base-pair alterations are on the same reads of the WES data through IGV. Although the patient had no aberrant MMR genes, nonsynonymous alterations on these driver genes might have triggered chromosomal instability and aberrant signaling pathways in ancestral tumor cells and initiated sporadic SIC from either tumor site B or E, which was in fair agreement with phylogenetic trees constructed by both tissues and PDOs.

With regards to the histological features of multifocal SICs, the staining patterns of subregional PDOs indicated that selective force derived from the organoid culture was not the major reason for this inconsistency. If the culture condition preferred certain types of subclones, all PDOs would exhibit unified staining patterns regardless of the tumor sites. Nevertheless, our data showed that PDOs from tumor sites C, E, and F had a moderate expression of both CDX2 and SMAD4, whereas PDOs from tumor sites A, B, and D rarely expressed both proteins. The site-specific SMAD4 mutation (K436M) was found in C-TO and F-TO, and both PDOs had moderate expressions of SMAD4, which suggested that this K436M alteration is not associated with loss of SMAD4.

The mutational distance of each tumor estimated with the phylogenetic analysis was somewhat different from the expression data. Hierarchical clustering analysis is based on Euclidean distance that captures the entire transcriptomic pattern of each tumor, whereas a phylogenetic tree is constructed using a Bayesian inference model with selected mutation data and its variant allele frequencies. Although some mutational traits such as splice-site variants were carried to the transcriptional level, there were other determinant factors contributing to the transcriptomic landscape. Besides, the weight of previously known driver mutations of the Cancer Gene Census largely affected the shape of trees. The differences between these analyses accounted for differential shapes between the phylogenetic tree and overall expressional patterns of synchronous tumors and their derivatives. Although the order of each tumor on the hierarchical clustering is not exactly matched to the phylogenetic tree, the innate clonal relationships were presented in multifocal SICs. For instance, the tumor sites B and D were adjacent to each other on both analyses, and tumor sites A and F were close to each other.

The sensitivity to HDAC inhibitors and demethylating agents reflected overall methylation profiles in PDOs. All tumor organoids were responsive to both belinostat and SAHA, inferring that inhibition of histone acetylase might have reactivated the control of cell growth and survival. Nevertheless, only C and F-TO were sensitive to azacitidine known to be incorporated into both DNA and RNA, which implied evidence of clonal differences in hypermethylation of tumor suppressor genes including *MLH1*, *MGMT*, and *FGFR2*. To investigate therapeutic targets in SIC, methylation of promoter regions should be considered as well as genetic alterations. Our study, nevertheless, lacked a direct comparison between the methylation status of original tumors and corresponding derivatives, which partially limited the clinical application.

In summary, our methodology offers a valuable preclinical resource that not only retained the majority of the histopathological characteristics of one specific SIC case but also reproduced the multiple layers of molecular clonality. We identified an unprecedented clonal relationship among multifocal CRCs with respect to the biological processes and drug responses as prominent phenotypes within a single patient. Our results also demonstrated heterogeneous drug sensitivities in multiple tumor masses within the bowel tract from the perspective of prevalent molecular heterogeneities. These data implied the imminent drawback of single biopsy-based PDOs when estimating patient responses to certain drugs and emphasized the significance of targeting progenitor somatic mutations mutually shared by all tumor masses to avoid unwanted drug resistance in SIC.

## Methods

### Ethics statement

The research protocol was reviewed and approved by the institutional review board of the Seoul National University Hospital (IRB No. 1102-098-357). The study was performed in accordance with the Declaration of Helsinki. Written informed consent was obtained from all patients enrolled in this study.

### Sample collection and preparation

A total of six tumor tissues with adjacent normal mucosa in both small and large bowels were obtained with written informed consent from Patient SNU-6685, who was diagnosed with synchronous intestinal cancer at the time of the surgical resection from Seoul National University Hospital (Seoul, Korea).

To be defined as synchronous, the tumors had to satisfy the following requirements: each tumor is histopathologically malignant and distinctively separated at least 4 cm from normal mucosa and other lesions; metastatic carcinoma or probability of metastasis from the other is excluded. This case was designated as synchronous tumors by Warren and Gate’s criteria of multiple primary cancer^39,40^. The synchronous tumors were resected from ascending colon, transverse colon, sigmoid colon, rectum, and small bowels, respectively, and designated as A–F according to their site of origin. Detailed clinical information about these six grossly separated tumors of Patient SNU-6685 is summarized in Table 1. The material was classified by adding “C” and “N” at the end of the letter representing “Cancer Tissue” and “Normal Tissue”. Normal as well as tumor tissues were cut into pieces and processed for immunohistochemistry and DNA/RNA extraction. The Tissue DNA was extracted using QIAamp Fast DNA Tissue Kit (Qiagen, Hilden, Germany) according to the manufacturer’s protocol. Total RNA was isolated from cell lysate using TRIzol (Qiagen, Hilden, Germany) and Qiagen RNeasy Kit (Qiagen, Hilden, Germany) according to the manufacturer’s protocol. The rest of the pieces were isolated for culture. Established derivatives included in the last character of the sample names: “TO”, “T”, and “Blank” indicate “Patient-Derived Tumor Organoid (PDOs)”, “Patient-Derived Tumor Organoid-Derived Cell Lines (PDOCs)”, “Patient-Derived Tumor Cell Line (PDCs)”, respectively. A living biobank of PDCs, PDOs, and PDOCs will be cryopreserved at Korean Cell Line Bank (KCLB, http://cellbank.snu.ac.kr) and distributed worldwide.

### Tumor isolation

The isolation and organoid culture of tumor tissues was performed as previously described by van de Wetering et al. ^23^ with a few adjustments. Tumors were chopped and incubated with Collagenase II (1.5 mg/ml), Hyaluronidase (20 μg/ml), and Ly27632 (10 μM) for 30 min at 37 °C shaking incubator. Basal culture medium (advanced Dulbecco’s Modified Eagle Medium/Ham’s F-12 supplemented with 1% (v/v) penicillin and streptomycin, 10 mM HEPES and Glutamax) with 10% FCS was added and the mixture was passed through a 100 μM cell strainer to remove debris or clumps. Cells were spun down at 1000 rpm for 3 min.

### Establishment of tumor-derived organoid culture

The dissociated tumor cells were resuspended in Basement Membrane Extract (BME) (Cultrex (R)PC BME RGF type 2, Amsbio) and plated the mixture into a flat-bottomed plate at different densities. After allowing the BME to be polymerized, the Human Intestinal Stem Cell medium (HISC) minus Wnt was added for tumor-derived organoid culture. HISC minus Wnt is composed of basal culture medium with 20% R-Spondin conditioned medium, 10% Noggin conditioned medium, 1 x B27, 1.25 mM n-Acetyl Cysteine, 10 mM Nicotinamide, 50 ng/ml human EGF, 10 nM Gastrin, 500 nM A83-01, 3 μM SB202190, 10 nM Prostaglandine E2 and 100 mg/ml Primocin (Vivogen). The isolated crypts from adjacent normal mucosa were also cultured with HISC minus Wnt, confirming that no normal organoid co-exists in established tumor organoids.

Culture medium was exchanged every 2 days. To passage the cultured organoids, BME was dissolved by pipetting and organoids were collected in a tube. The organoids were centrifuged at 1000 rpm for 3 min and the medium are removed. Five ml of TrypLE Express (Invitrogen) was added and the organoids were incubated at 37 °C for ~5 min. FCS and medium were added and cells were spun down at 1500 rpm for 3 min. The pellet was taken up in BME and cells were plated in droplets of 50–100 μL each. After allowing the BME to solidify, HISC minus Wnt supplemented with 10 μM LY27632 was added to the plates.

### Establishment of tumor-derived cell line and tumor organoid-derived cell line culture

The isolated tumor cells were resuspended in Opti-MEMI (Thermo Fisher Scientific, MA, USA) with 5% fetal bovine serum (FBS) and seeded into T-25 cm^2^ or T-75 cm^2^ flasks. Trypsinization in a confined space or scraping method was used to obtain pure tumor cells when stromal cells grew in the initial culture.

With respect to the characteristics of epithelial cells and their barrier, synchronous intestinal organoid-derived cell lines were devised and cultured in 2D monolayer to overcome the impermeability of the luminal side of organoids^41^. Tumor organoid-derived cell lines originated from six established organoids. After removal of the culture medium, organoids were broken up by pipetting with 5 ml of TrypLE Express. Dissolved organoids were collected in a tube and incubated at 37 °C for ~5 min. FCS and medium were added and cells were spun down at 1500 rpm for 3 min. Dissociated cells were resuspended in basal culture medium with 20% R-Spondin conditioned medium, 10% Noggin conditioned medium, and seeded into T-25 cm^2^ or T-75 cm^2^ flasks. After primary culture, established cancer cell lines and organoid-derived cell lines were sustained in RPMI 1640 (Thermo Fisher Scientific, MA, USA) with 10% fetal bovine serum and 1% (v/v) penicillin and streptomycin (10,000 U/ml). Incubated flasks in humidified incubators at 37 °C in an atmosphere of 5% CO_2_ and 95% air.

### Establishment of patient-derived organoid xenograft

All animal experiments were conducted with approval from the Institutional Animal Care and Use Committees (IACUC) of the Korea Research Institute of Bioscience and Biotechnology (KRIBB-AEC-2118). They were carried out in accordance with institutional guidelines and regulations (https://www.alio.go.kr/popSusiView21110.do?seq=2016102601282472. All animals were maintained with free access to food and water in a specific-pathogen-free facility under a 12 h–12 h light–dark cycle (lights on at 7:00; lights off at 19:00) with a temperature of 22 ± 0.5 C and humidity of 55 ± 15%. Each established SIC organoid was transplanted into six male nude mice independently. Mice were anesthetized by intraperitoneal administration of a 2.4% Evertin solution at 300 μL/mouse. Implantation of tumor organoids was conducted at a heating plate set at 35 °C. A total 100 μL of organoid suspension was injected into the subcutaneous pouch of an anesthetized mouse with a 200 μL pipette and the dissection was closed with a surgical clip. Anesthetized mice were observed until awakening from anesthesia, and returned to the mouse cage. The size of the tumors on both left and the right side was measured and recorded once a week. The mice were observed up to 10 weeks of age, and then autopsies were performed. Tumors were formalin-fixed immediately after autopsy and embedded in paraffin for IHC analysis.

### H&E staining and immunohistochemistry

Tumor specimens were embedded in 10% neural buffered formalin and fixed in paraffin. Then paraffin blocks were sliced at 4 µm thickness. Organoids were mechanically dissociated from the BME dome by repeated centrifugation. Organoids were then embedded in 2% agarose gel (INTRON Biotechnology, Seongnam, Korea). Solidified agarose gel was fixed in 10% formalin for 30 min at room temperature and sectioned at 4 µm thickness. Both tissue and organoid sections were subjected to H&E as well as immunohistochemical (IHC) staining. The procedure of IHC is as previously described^42^. Antibodies used for IHC are summarized as follows:

**Table:** 

Target	Company	Catalog; RRID	Dilution
Mouse monoclonal anti-Cytokeratin 20	Santa Cruz Biotechnology	Cat# sc-271183; RRID:AB_10610054	1:200
Mouse monoclonal anti-CDX2	BioGenex	Cat# AM392; RRID:AB_2650531	1:300
Mouse monoclonal anti-β-catenin	BD Biosciences	Cat# 610153; RRID:AB_397554	1:800
Rabbit polyclonal anti-KI-67	Abcam	Cat# ab15580; RRID:AB_443209	1:500
Mouse monoclonal anti-MLH1	Santa Cruz Biotechnology	Cat# sc-56161; RRID:AB_784582	1:500
Mouse monoclonal anti-MSH2	Santa Cruz Biotechnology	Cat# sc-137015; RRID:AB_2144968	1:500
Mouse monoclonal anti-MSH6	Santa Cruz Biotechnology	Cat# sc-271080; RRID:AB_10611658	1:500
Mouse monoclonal anti-PMS2	Santa Cruz Biotechnology	Cat# sc-25315; RRID:AB_628163	1:500
Mouse monoclonal anti-SMAD4	Santa Cruz Biotechnology	Cat# sc-7966; RRID:AB_627605	1:500

### Whole-exome sequencing

Total DNA was isolated from the cell lines and organoid pellet using QIAamp DNA Mini Kit (Qiagen, Hilden, Germany) according to the manufacturer’s protocol. The Tissue DNA was extracted using QIAamp Fast DNA Tissue Kit (Qiagen, Hilden, Germany) according to the manufacturer’s protocol. Whole-exome capture was performed on all samples including normal colon mucosa tissue with the SureSelect Human All Exon V5 Kit (Agilent Technologies, Tokyo, Japan). The captured targets were subjected to sequencing using HiSeq 2500 (Illumina, San Diego, CA, USA) with the pair-end 100 bp read option for organoid samples and 200 bp read option for tissue materials. The sequence data were processed through an in-house pipeline. Briefly, paired-end sequences were aligned to the human reference genome (UCSC assembly hg19—original GRCh37 from NCBI, 2009) using the mapping program BWA (version 0.7.12)^43^, and generated a mapping result file in BAM format using BWA-MEM. PCR duplicates were removed using MarkDuplicates.jar included in Picard tools (v. 1.130, https://broadinstitute.github.io/picard/). The Genome Analysis Toolkit (GATK, v.3.4.0)^44^ was used to perform base quality score recalibration (BQSR) and local realignment around indels. Somatic mutations were identified by providing sequence alignment data of tumor and normal to the MuTect (involved in GATK v3.8.0) with default parameters using tumor–normal mode taking both SNVs and short indels into account. We used hg19 as a reference genome build. Those variants are annotated by SnpEff v4.1g, to vcf file format, annotating with dbSNP for the version of 142 and SNPs from the 1000 genome project. Then, SnpEff was applied to annotate additional databases, including ESP6500, ClinVar, dbNSFP 2.9. Mutational signatures were calculated using the MutationalPatterns R package v.3.4.0^45^ to detect distinct footprints in the genomic context of somatic SNVs and evaluate mutational mechanisms in the six tumor tissues and matched cell lines/organoids. The absolute contribution of mutational patterns designated multiple mutational signatures per sample. The algorithm estimating the absolute contribution of substitution patterns was to fit 96 types of substitution into the previously constructed mutational signature. Therefore, a single substitution can be assigned to multiple signatures, which is likely to be interpreted to other signatures.

### Analysis of CNVs

For the detection of Copy Number Variations (CNVs) and loss of heterozygosity (LOH) from exome sequencing data, we employed ExomeCNV package in R program^46^. We first calculated the log ratio of the depth of coverage between paired tumor and normal samples. The ratios are normalized by the total number of reads and adjusted so that the median log ratio of exons on “normal” chromosomes is zero. Then, we calculated the specificity and sensitivity (power) of detecting CNV based on the depth of coverage and log ratio of all exons. We made a call when sufficient specificity and sensitivity are achieved. Next, we calculated log ratios and called segments using exon midpoints as the probe positions. CNV calls were expressed as 1, 2, and 3 which indicated deletion, normal, and amplification, respectively.

### Construction of evolutionary trees

The evolutionary trajectory of synchronous intestinal cancers was constructed by Treeomics v1.9.2 algorithm^19^ using whole-exome sequencing data. Treeomics setting was as follows: sequencing error rate = 0.005, prior absent probability = 0.5, max absent VAF = 0.05, LOH frequency = 0, false discovery rate = 0.05, false-positive rate = 0.005, and absent classification minimum coverage 100. Input parameters include the number of variant reads, the number of total reads, gene symbols, chromosomal coordinates, and substitutional patterns.

Treeomics highlights any non-synonymous or splice-site variants in putative driver genes given in a CSV file. As a default list, the union of reported driver genes by 20/20+, TUSON, and MutsigCV^47^ was used. As an optional input, we adapted Cancer Gene Census (CGC) annotation that further checks input variants if they are listed on the provided CSV file (CGC version 80, reference genome hg19). Variants presented in both the default list and the CGC list were highlighted in the inferred phylogeny. MUC6 mutations outnumbered those found in other genes as several studies have already suggested that the MUC6 gene should be considered with skepticism when predicting pathogenicity based on WES results due to the extremely high local polymorphism^48–50^. Eventually, MUC6 mutations highly shaped the branches of the phylogenetic trees. To avoid unwanted statistical weight from a single gene, we purposely omitted MUC6 mutations from the input data. Treeomics program adjusted sequencing artifacts to check if the mutational patterns were in parallel with the topologic structure of the phylogenetic trees. Sub-clonal analysis was additionally performed by adding “-u” parameter to input commands.

### Analysis of RNA sequencing

Total RNA was isolated from cell lysate using TRIzol (Qiagen, Hilden, Germany) and Qiagen RNeasy Kit (Qiagen, Hilden, Germany). Paired-end sequencing reads from cDNA libraries (101 bp) were generated with an Illumina NovaSeq6000 instrument and the sequence quality was verified with FastQC v.0.11.7 (https://www.bioinformatics.babraham.ac.uk/projects/fastqc/). For data preprocessing, low-quality bases and adapter sequences in reads were trimmed using Trimmomatic v 0.38^51^. The trimmed reads were aligned to the human genome (UCSC hg19) using HISAT v2.1.0, a splice-aware aligner^52^. Then, transcripts including novel splice variants were assembled with StringTie v1.3.4d^53^. The abundance of these transcripts in each sample was calculated as read counts or TPM (Transcript per Million mapped reads) values. For each sample, the transcript expressions were normalized by dividing read counts into lengths of the mapped genes. These normalized transcript values (transcripts per million, TPM) of 35,993 genes were divided by the values of normal colon mucosa and transformed to log_2_-fold change to investigate the differential transcriptome between multiple sites.

### Principle component analysis

To perform principal components analysis and confirm the similarity distance among the samples, the dist and prcomp functions were used from the ggdendro (v0.1.22) and ggfortify (v0.4.11) R package, respectively. Hierarchical clustering analysis was conducted using the log2-transformed values of 35,993 genes, and the samples were clustered into 5 subgroups by the fviz_dend function from factoextra (v1.0.7) R package. Then, the first PCA plots were calculated using the normalized transcripts values of PDO group. The second PCA plot was determined in the 11 gene sets (759 genes) of KEGG database among synchronous tumors and corresponding PDOs. To access the internal data of gene loadings and analyze the contributing variables of PC1 which split the samples by specific tumor sites, the loading components were calculated by advanced features of the pca function from PCAtools (v1.2.0) R package. The 20 highest loading components containing both positive and negative values in PC1 were plotted in a bar graph.

### Enriched pathway analyses on mRNA level

Based on the raw read counts of 35,993 transcripts, a single sample enrichment level analysis of cell signaling pathway was conducted by GSEA(v4.1.0) using hosted MSigDB gene set database of KEGG library (c2.cp.kegg.v7.4.symbols.gmt). The phenotype label was designated as either normal colon mucosa versus tumor tissue or versus corresponding tumor derivatives for normalized enrichment score(NES) of a single sample. Independent NES of the paired sample (normal and tumor) was calculated on default fields with the setting of permutations as 1000 and phenotype. The result was annotated by NCBI Gene ID MsigDB.v7.4.chip platform. Total of overlapped pathways (50 gene sets of KEGG pathway) which were significantly different (FDR < 0.25) compared to normal control in both multiple tumors and PDOs were selected to identify the recapitulations of the differentially expressed pathway in derived models. Using ComplexHeatmap (v2.2.0) R package, heatmap of NES values was plotted and the mapped color variance was set between the minimum and maximum value.

For cnetplot, the top 5% contributing factors (*n* = 3598) within the loading components of PC1, which showed both negative and positive correlations, were mapped with a genome-wide annotation database for humans using org.Hs.eg.db (v3.10.0) R package. Then, annotated genes were transformed to Entrez ID for Reactome pathway over-presentation analysis using ReactomePA (v1.30.0) R package^54^. Only those pathways which were associated with mapped genes were selected by the enrichPathway function with Benjamini–Hochberg correction and a cutoff (*p* < 0.05, *q* < 0.2). Then, the gene-concept network categorized by involved genes ratio was visualized with the function cnetplot from DOSE (v3.12.0) R package.

### Analysis of methylation sequencing

The capture libraries for targeted methylation sequencing were prepared according to the manufacturer’s instructions of SureSelectXT Methyl-Seq Library Preparation kit (Agilent Technologies, Germany) and Methyl-Seq Target Enrichment System for Illumina Multiplexed Sequencing Protocol; Version E0, April 2018 (Agilent manual Part Number G7530-90002). Briefly, fragmentation of 3 μg of genomic DNA was performed using the Covaris LE220 focused-ultrasonicator (Covaris, Woburn, MA) to a target peak size of 150–200 bp. The fragmented DNA is repaired, an ‘A’ is ligated to the 3’ end, SureSelect Methyl-Seq Methylated Adapter is then ligated to the fragments. According to the manufacturer protocol, the methylated adapter-ligated DNA is then quantified using the TapeStation DNA screentape D1000. For standard SureSelectXT Methyl-Seq target enrichment, 350 ng of DNA library was mixed with hybridization buffers, blocking mixes, RNase block, and 5 µl of SureSelect Methyl-Seq Capture Library. Hybridization was conducted at 65 °C using heated thermal cycler lid option at 105 °C for 24 h on PCR machine. The captured DNA was then washed and amplified. The final purified product was quantified using qPCR according to the qPCR Quantification Protocol Guide (KAPA Library Quantification kits for Illumina Sequencing platforms, KR0405, Version 9.17) and qualified using the TapeStation DNA screentape D1000 (Agilent). And then we sequenced using the HiSeq platform (Illumina, San Diego, USA).

### Concordance of epigenetic patterns within PDOs

The extents of methylation compared to the normal mucosa samples were calculated by subtracting the value of the organoid sample with paired normal control. Overlapping methylated promoter sites (129,807 positions) were analyzed to identify the correlation between samples. Then, only 14,434 promoter regions in PDOs were selected by PANcancer related geneset (299 genes) which were involved in tumor-suppressing processes. To assess the concordance of subregional clones among PDOs, the matrix of Pearson’s correlation coefficient was computed with the function round and signify from base (v3.6.2) R package. The heatmap of the correlation matrix was plotted by ComplexHeatmap R package. Hierarchical clustering of epigenetic patterns among PDOs was conducted with the same method as RNA-seq analysis, but *k*-mean clustering grouped the samples into 3 subgroups.

### Enriched pathway analyses on epigenome (ssGSEA)

Only the mapped methylated promoter gene ID (122,780 sites) from Entrez ID database were analyzed for GSEA analysis of epigenome among PDOs. Methylated values in the region of promoter gene of PDO versus normal controls were analyzed for differential epigenetic changes compared to normal mucosa. The rest of the analysis fields were identical to GSEA analysis on mRNA level. Total of 14 pathways (FDR < 0.25) in gene sets of the KEGG database overlapped within PDOs. Heatmap of NES values indicated the extent of distinctively methylated in the promoter regions which were involved in the gene set of cellular essential processes and ranged from -4(blue, hypo-methylated) to 4(red, hyper-methylated).

### Differential epigenetic alteration

Based on the result of concordance patterns, PDOs were categorized into two clustered organoid pairs which were C with F-TO, and the rest of PDOs to investigate the significantly distinct epigenome. Within 14,434 methylated sites in promoter regions, only those methylation values (*m*) were selected only if the standard deviation (SD) of the values among PDOs was over 0.1. The methylation patterns were divided into 2 conditions whether C and F-TO were hyper- or hypo-methylated. By setting the cutoff as the variation (SD < 0.1) between the *m* value of C and F-TO, 63 and 54 promoter sites were sorted in the state of C/F-TO hypermethylation (*m* > or =0) and hypomethylation (*m* < 0), respectively. The heatmap of differential epigenetic alteration was plotted and the color variance ranged from −1(hypomethylated) to 1(hypermethylated).

### 2D organoids seeding/treatment procedure

2 × 10^5^ to 4 × 10^5^ viable cells from each cell line were seeded into well of 96-well white plate (SPL, #30196) in triplicate to measure the sensitivity of several drugs. Complete Human Intestinal Stem Cell (HISC) medium through the entire procedure for drug screening to minimize the possible variables from using different culture mediums. The following day all cell lines were treated with proper concentrations of the 25 compounds.

### 3D organoids seeding/treatment procedure

All drug screens were performed two times. Organoids were mechanically and enzymatically dissociated into single cells by incubating in TrypLE (Gibco) for 5–10 min. The suspension (5 μl/well) was dispensed in clear-bottomed, white-walled 96-well plates (#3903, Corning) using an automated repeat pipet and overlaid with 200 μl of a 1:1 mixture of HISC medium and RGF basement membrane matrix (Gibco, A14132-02). Plates are incubated at 37 °C with 5% CO_2_ for 15 min to solidify the gel before the addition of 20 µl of pre-warmed HISC medium to each well using an EpMotion (Eppendorf). 96 h after seeding, 20 µl of the drug-containing solution is added to each well. For the control well, the mixture of HISC medium and the drug-solvent solution is added.

### ATP detection assay and statistical analysis of drug response

After 72 h of drug treatment, 10 µl of Celltiter-Glo 2D (Promega #G9241) and 3D Reagent (Promega #G968B) is added to each well of the cancer cell line and organoid, respectively, followed by 5 min of vigorous shaking. After 30-min incubation at room temperature and an additional minute of shaking, luminescence is measured with a Luminoskan Ascent (Thermo Scientific) over 1000 ms of integration time. The ATP detection level from vehicle-treated samples was used for normalization. For two methyltransferase inhibitors, azacitidine and decitabine, EC_50_ values for each subregional organoid were estimated using GraphPAD Prism 7 for Windows (GraphPad Software, La Jolla, CA, USA). The responses of the other drugs were calculated as AUC values and visualized with *k*-means clustered heatmap from ComplexHeatmap package (v. 2.13.0) from R program version 4.2.0 (R Foundation for Statistical Computing, Vienna, Austria). Optimal number of clusters for derivatives and drugs was calculated with factoextra package (v. 1.0.7) with the elbow method from R program (R Foundation for Statistical Computing). For hierarchical cluster analysis on a set of dissimilarities, each object was assigned to its own cluster, which an algorithm proceeds through iteratively. Two of the most similar clusters are joined at each stage until there is a single cluster. Euclidian distances between clusters are recomputed at each stage by the Lance–Williams dissimilarity update formula according to the single linkage method. For comparing drug responses among multiple clones, two-way ANOVA with Bonferroni post-test was performed using GraphPad Prism version 7 for Windows (GraphPad Software).

### Multi-omics integration

The drug response data is integrated with RNA-seq and methyl-seq data using mixOmics R Bioconductor package^55^ with built-in analyzing and visualization functions. We have limited the construction matrix to 3 parameters: RNA-seq (759) × methyl-seq (328) × drug responses (25) estimating more than six million multi-omics combinations. We focused on the expression patterns and promoter methylation status of tumorigenesis-related genes by referring to previously reported pan-cancer driver genes^7^. Detailed code is described in the code availability section. We first built a pseudo-design matrix utilizing multiblock partial least squares (PLS)- discriminant analysis (DA) to identify correlated variables across three different input datasets. Each numeric value from expression, methylation, and drug responses (latent components or linear combinations of variables) was constructed such that the sum of covariances between all pairs of datasets is maximized. Then, we estimated the relationship structure between the various inputted data, where each value (between 0 and 1) represents the strength of the relationship among three given data-frames. All pairwise covariances were weighted as indicated by the design matrix. The response variable is transformed into a dummy variable (i.e. ‘one hot encoded’) internally within the function. The regression sparse generalized canonical correlation analysis (sGCCA) framework from the RGCCA package is utilized to deflate each of the datasets. The transformed variables were combined for further discrimination and integration.

### Reporting summary

Further information on research design is available in the Nature Research Reporting Summary linked to this article.

## Supplementary information

Supplementary Information

Supplementary Data 1

Supplementary Data 2

Supplementary Data 3

Supplementary Data 4

Supplementary Data 5

Nature Reporting Summary

## Data Availability

Cell lines and organoids generated in this study have been deposited to the KCLB (Korean Cell Line Bank, https://cellbank.snu.ac.kr) biobank and are governed by Ja-Lok Ku. Raw next-generation data of all DNA, RNA, and epigenetic sequencing is deposited to Genome Sequence Archive (GSA) for Human with an accession number of HRA002557 in accordance with the MINSEQE standards. Raw and processed sequencing data will be available by request and governed by the Lead Contact.

## Code availability

Computational pipelines as well as sequencing data including whole-exome sequencing and RNA-sequencing in this study are available at the public repository (https://github.com/nahyun828/SIC).
