# Supplementary material for: Multifocal organoids reveal clonal associations between synchronous intestinal tumors with pervasive heterogeneous drug responses
Source: NPJ Genom Med. 2022 Jul 19;7:42. doi: 10.1038/s41525-022-00313-0 (PMC9296490; doi:10.1038/s41525-022-00313-0)
Supplement: Supplementary file 1 — Supplementary Information [file 41525_2022_313_MOESM1_ESM.pdf]

Supplementary Information

Multifocal Organoids Reveal Clonal Associations between Synchronous Intestinal Tumors with Pervasive Heterogeneous Drug Responses

Nahyun Jeong[1,2,9], Soon-Chan Kim[1,2,3,4,9], Ji Won Park[2,5,6], Seul Gi Park[7], Ki-Hoan Nam[7], Ja Oh Lee[1,2], Young-Kyoung Shin[1,2,4], Jeong Mo Bae[8], Seung-Yong Jeong[2,5,6], Min Jung Kim[2,5,6,*], and Ja-Lok Ku[1,2,3,4,10,*]

[1]Korean Cell Line Bank, Laboratory of Cell Biology, Cancer Research Institute, Seoul National University College of Medicine, Seoul 03080, Korea
[2]Cancer Research Institute, Seoul National University, Seoul 03080, Korea
[3]Department of Biomedical Sciences, Seoul National University College of Medicine, Seoul 03080, Korea
[4]Ischemic/Hypoxic Disease Institute, Seoul National University College of Medicine, Seoul, 03080, Korea
[5]Department of Surgery, Seoul National University College of Medicine, Seoul 03080, Korea
[6]Division of Colorectal Surgery, Department of Surgery, Seoul National University Hospital, Seoul 03080, Korea
[7]Laboratory Animal Resource Center, KRIBB, Chungbuk 28116, Korea
[8]Department of Pathology, Seoul National University College of Medicine, Seoul 03080, Korea
[9]These authors contributed equally
[10]Lead contact

**Correspondence:*

Min Jung Kim, Department of Surgery, Seoul National University College of Medicine, 103, Daehak-ro, Jongno-gu, Seoul 03080, Korea
E-mail: minjungkim@snuh.org

Ja-Lok Ku, Laboratory of Cell Biology, Cancer Research Institute, Seoul National University College of Medicine, 103, Daehak-ro, Jongno-gu, Seoul 03080, Korea
E-mail: kujalok@snu.ac.kr

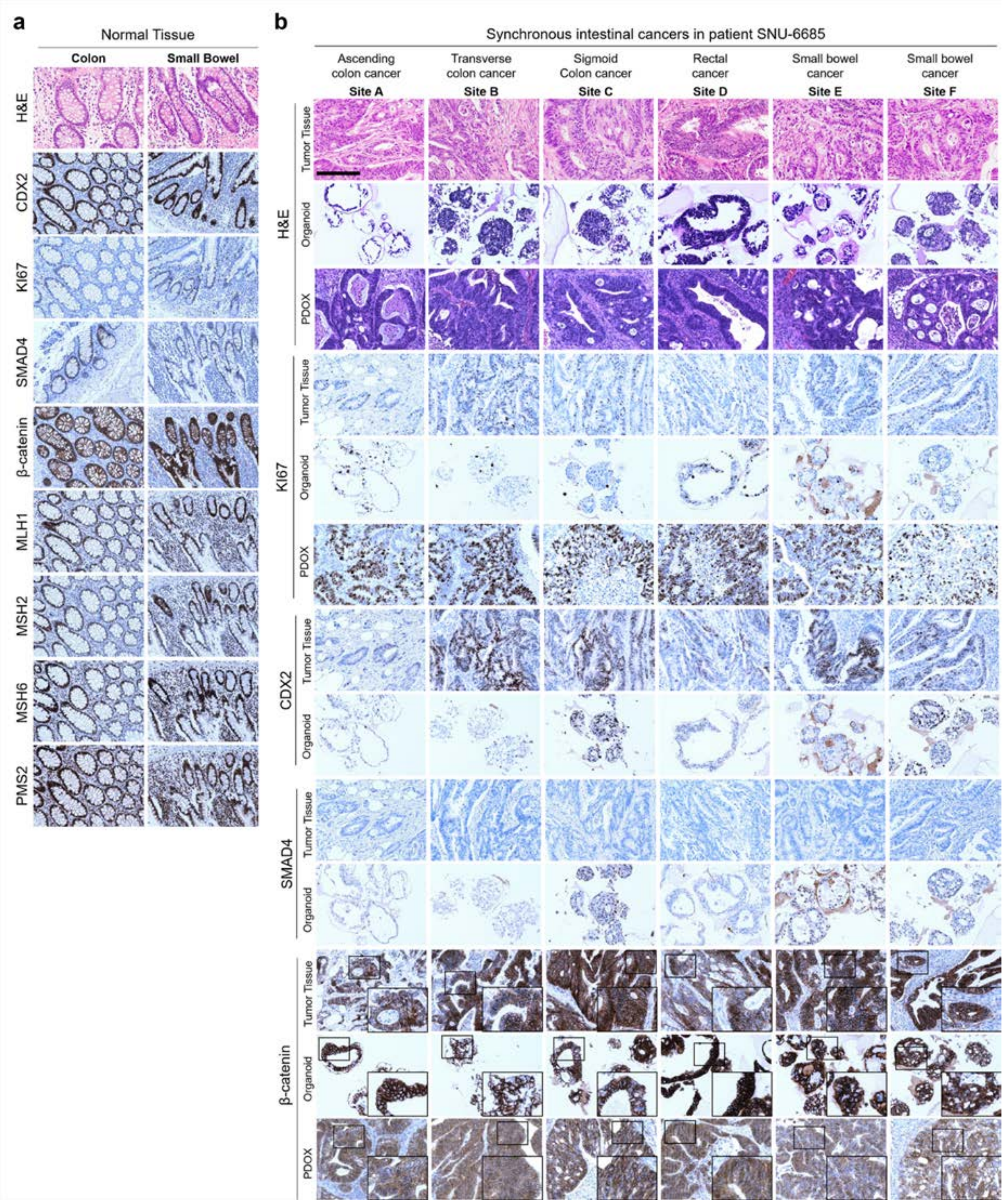

Supplementary Fig. 1a and 1b. Organoids as well as patient derived organoid xenograft resembles primary tumor epithelium in histological features. Immunohistochemistry of 6 established patient derived tumor organoids (PDO), PDO derived xenograft(PDOX) and their corresponding tumor tissues with 2 normal small and large bowel mucosa. Hematoxylin-eosin stain(H&E) staining on primary tumors and the corresponding organoids revealed that the "cystic versus solid" organization of the epithelium was generally preserved. Specific marker expression analysis was conducted using β-catenin, KI67, Caudal type homeobox2 (*CDX2*), and SMAD family member 4 (*SMAD4*). Expression of these diagnostic markers revealed that individual PDOs and PDOXs resembled their corresponding tumors both in the pattern and heterogeneity of the expression, Scale bar = 200µm and *insets* are X400.

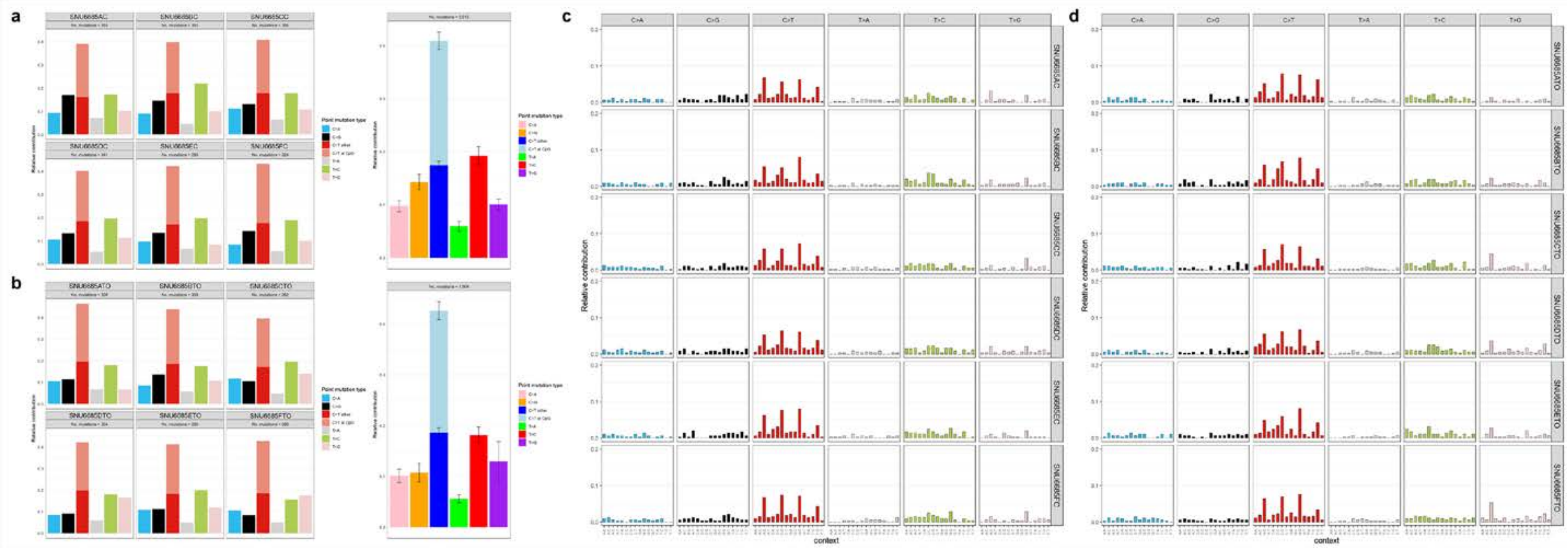

Supplementary Fig. 2a and 2b. The most predominant point mutation type in both tissue specimens and PDOs was C-to-T transitions, and the pattern of distribution was analogous. The error bars represent the standard deviations of relative contribution of point mutations detected in tumors ($n = 6$) and their derivatives ($n = 6$) respectively. **2c and 2d.** Relative contribution was highly concordant between tissues and organoids.

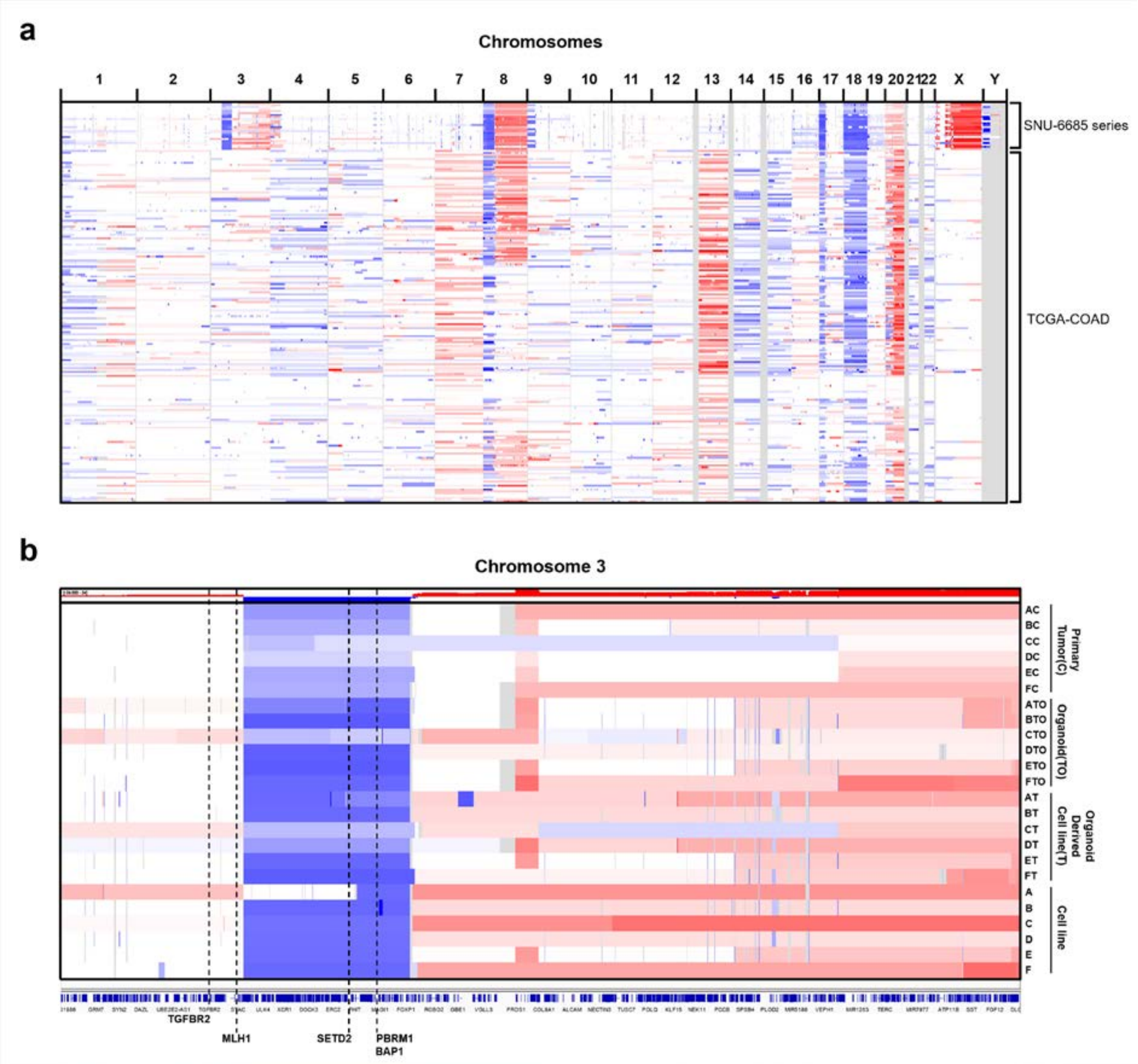

Supplementary Fig. 3a. Genome-wide copy number alteration map identified specific genomic aberration on chromosome 3 compared to the TCGA-COAD colorectal cancer cohort. **3b.** Several genes involved in epigenetic regulations on chromosome 3 were lost in all multiple tumors and their derivatives.

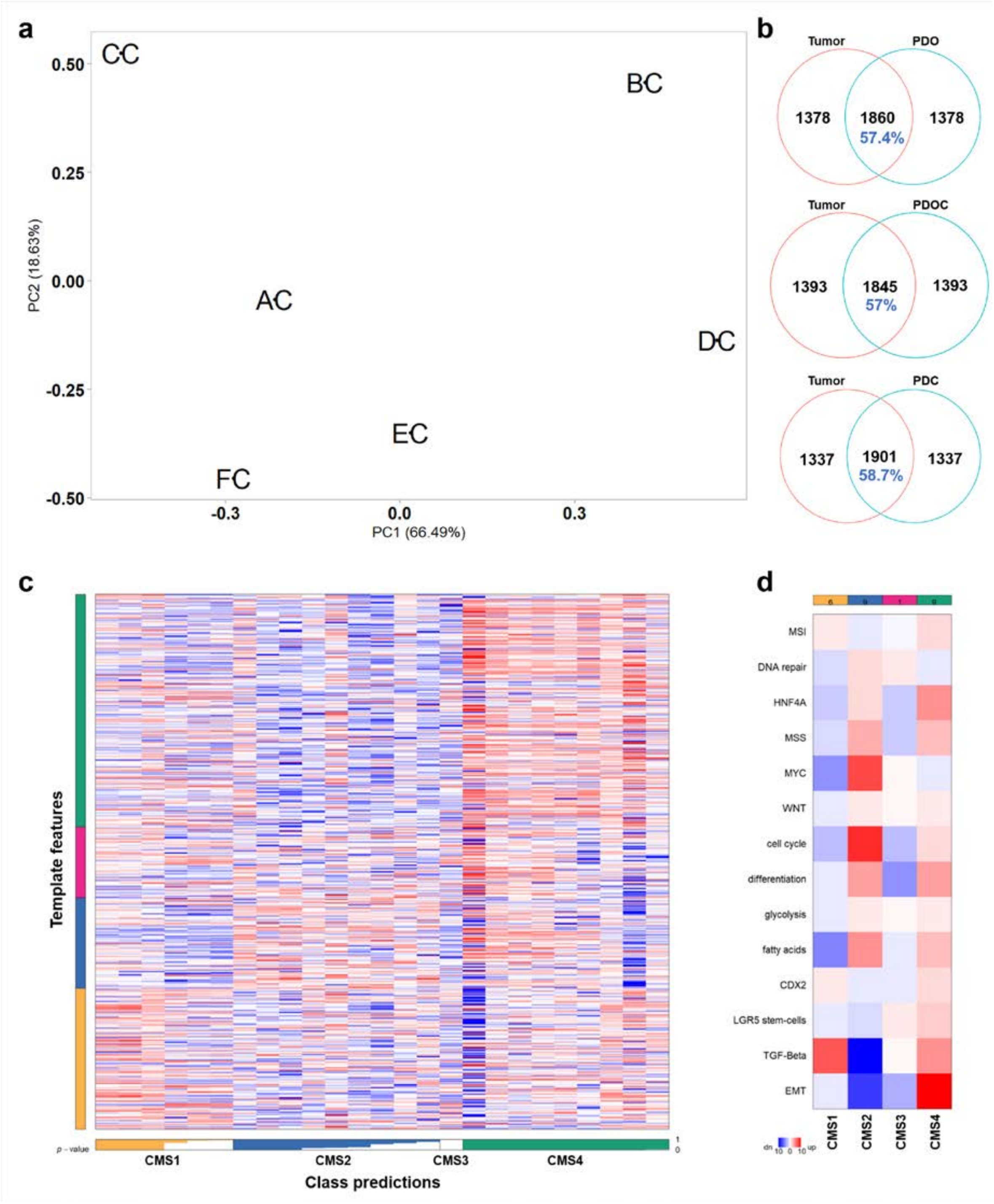

Supplementary Fig. 4a. Principle Component Analysis (PCA) indicating intertumoral heterogeneity of transcriptome of synchronous tumors. The PC1 is indicated on the x-axis and PC2 is indicated on the y-axis with composing percentages. **4b.** Venn diagram showing overlaps of top 10% of transcriptomes contributing to PC1 of the original tumors and their derivatives including PDOs, PDOCs and PDCs respectively. **4c.** Consensus molecular subtyping of multifocal tumors (*n* = 6) and corresponding derivatives (*n* = 18). Class predictions are shown in x-axis and template features are exhibited in y-axis with the representative color of each subtype (red, upregulated; blue, downregulated). **4d.** Heatmap showing scores in 14 representative pathways (red, upregulated; blue, downregulated).

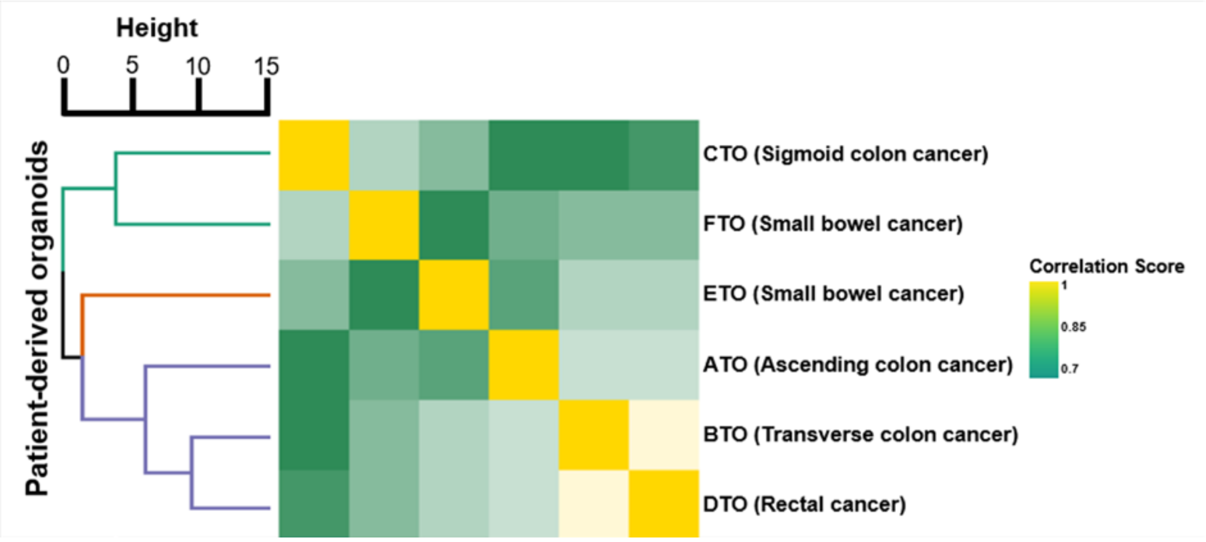

Supplementary Fig. 5. 14,434 methylated promoter sites occurred in PDOs were selected by PANcancer related geneset (299 genes) to compare differential methylation profiles.

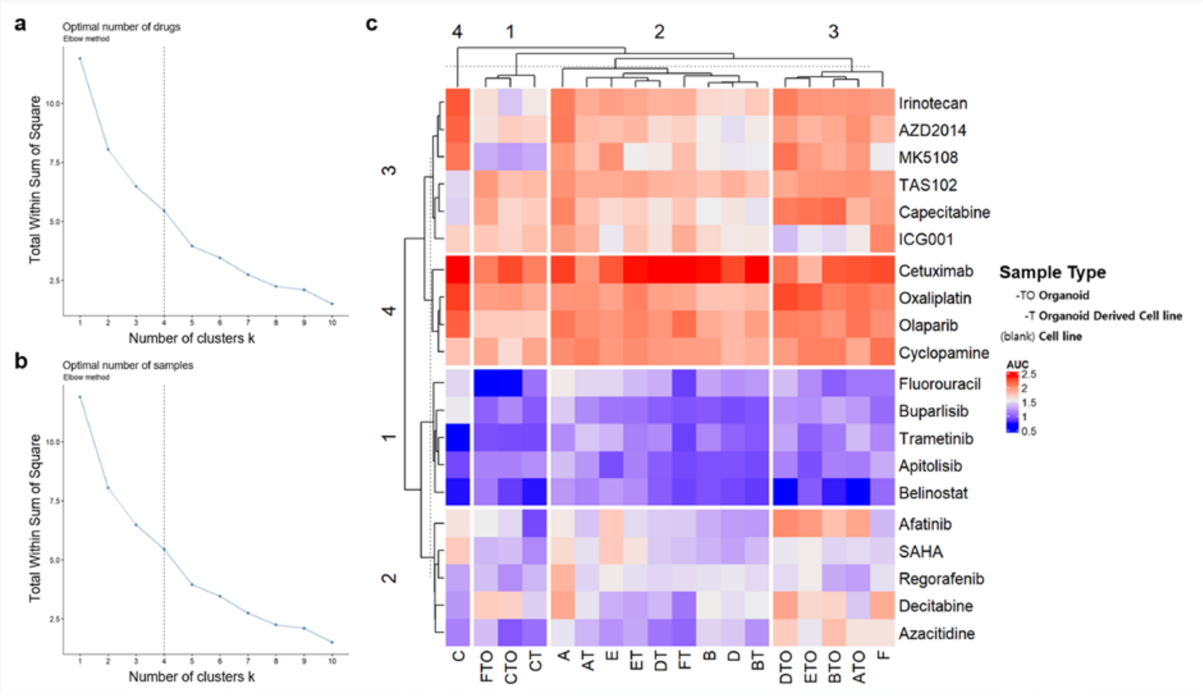

Supplementary Fig. 6a and 6b. Elbow method indicated that there are 4 optimal clusters in both drugs and derivatives. **6c.** K-means clustering AUCs according to number of clusters from the elbow method identified three major sub-groups among the screened compounds.
