# Supplementary material for: Multifocal organoids reveal clonal associations between synchronous intestinal tumors with pervasive heterogeneous drug responses
Source: NPJ Genom Med. 2022 Jul 19;7:42. doi: 10.1038/s41525-022-00313-0 (PMC9296490; doi:10.1038/s41525-022-00313-0)
Supplement: Supplementary file 7 — Nature Reporting Summary [file 41525_2022_313_MOESM7_ESM.pdf]

nature portfolio

Corresponding author(s): Min Jung Kim and Ja-Lok Ku

Last updated by author(s): Jan 14, 2022

Reporting Summary

Nature Portfolio wishes to improve the reproducibility of the work that we publish. This form provides structure for consistency and transparency in reporting. For further information on Nature Portfolio policies, see our Editorial Policies and the Editorial Policy Checklist.

Statistics

For all statistical analyses, confirm that the following items are present in the figure legend, table legend, main text, or Methods section.

n/a	Confirmed	
☐	☒	The exact sample size (*n*) for each experimental group/condition, given as a discrete number and unit of measurement
☐	☒	A statement on whether measurements were taken from distinct samples or whether the same sample was measured repeatedly
☐	☒	The statistical test(s) used AND whether they are one- or two-sided<br>*Only common tests should be described solely by name; describe more complex techniques in the Methods section.*
☐	☒	A description of all covariates tested
☐	☒	A description of any assumptions or corrections, such as tests of normality and adjustment for multiple comparisons
☐	☒	A full description of the statistical parameters including central tendency (e.g. means) or other basic estimates (e.g. regression coefficient) AND variation (e.g. standard deviation) or associated estimates of uncertainty (e.g. confidence intervals)
☐	☒	For null hypothesis testing, the test statistic (e.g. *F*, *t*, *r*) with confidence intervals, effect sizes, degrees of freedom and *P* value noted<br>*Give P values as exact values whenever suitable.*
☒	☐	For Bayesian analysis, information on the choice of priors and Markov chain Monte Carlo settings
☐	☒	For hierarchical and complex designs, identification of the appropriate level for tests and full reporting of outcomes
☐	☒	Estimates of effect sizes (e.g. Cohen's *d*, Pearson's *r*), indicating how they were calculated

Our web collection on statistics for biologists contains articles on many of the points above.

Software and code

Policy information about availability of computer code

Data collection: N/A

Data analysis:

Whole-exome sequencing
Whole-exome capture was performed on all samples including normal colon mucosa tissue with the SureSelect Human All Exon V5 Kit (Agilent Technologies, Tokyo, Japan). The captured targets were subjected to sequencing using HiSeq 2500 (Illumina, San Diego, CA, USA) with the pair-end 100 bp read option for organoid samples and 200 bp read option for tissue materials. The sequence data were processed through an in-house pipeline. Briefly, paired-end sequences are firstly mapped to the human genome, where the reference sequence is UCSC assembly hg19 (original GRCh37 from NCBI, Feb. 2009) using the mapping program BWA (version 0.7.12), and generated a mapping result file in BAM format using BWA-MEM. Then, Picard-tools (ver.1.130) were applied in order to remove PCR duplicates. The local realignment process is performed to locally realign reads with BAM files reducing those reads identically match to a position at start into a single one, using MarkDuplicates.jar, which requires reads to be sorted. By using Genome Analysis Toolkit, base quality score recalibration (BQSR) and local realignment around indels were performed. Haplotype Caller of GATK (GATKv3.4.0) was used for variant genotyping for each sample based on the BAM file previously generated (SNP and short indels candidates are detected). Somatic mutations were identified by providing the reference and sequence alignment data of tumor tissues or organoids to the MuTect2 (involved in GATK v3.8.0) with default parameters using tumor-normal mode. Those variants are annotated by SnpEff v4.1g, to vcf file format, filtering with dbSNP for the version of 142 and SNPs from the 1000 genome project. Then, SnpEff was applied to filter additional databases, including ESP6500, ClinVar, dbNSFP 2.9. Mutational signatures were evaluated using the Mutational Patterns R package, release 3.6.1 to configure distinct footprints in genomic context for all somatic SNVs and evaluate a multitude of mutational patterns in base substitution in 6 tumor tissues and matched cell lines/organoids.

Analysis of CNVs
For the detection of Copy Number Variations (CNVs) and loss of heterozygosity (LOH) from exome sequencing data, we employed ExomeCNV package in R program. The final log ratio of depth of coverage was determined by the number of bases targeted by exome sequencing (targeted base) and the number of bases actually sequenced (mapped). CNV calls were expressed as 1, 2, and 3 which indicated deletion,

normal and amplification respectively.

Construction of Evolutionary Trees
Evolutionary trajectory of synchronous intestinal cancers was constructed by Treeomics algorithm using whole exome sequencing data. Treeomics setting was as follows: sequencing error rate = 0.005, prior absent probability = 0.5, max absent VAF = 0.05, LOH frequency = 0, false discovery rate = 0.05, false-positive rate = 0.005, and absent classification minimum coverage 100. Input parameters include read depths of both mutant and coverage genes, gene symbols, chromosomal coordinate, and substitutional patterns.
Treeomics algorithm also provided likely driver gene mutations and built-in Cancer Gene Census List. The number of mutations harboring MUC6 gene outnumbered other genes. Consequently, the structure of the evolutionary tree was largely affected by the MUC6 variations. To eliminate potential bias, we excluded mutations of MUC6 genes from the input data. Sequencing artifacts were automatically adjusted by Treeomics default setting to confirm the topologic configuration of the evolutionary tree was compatible with the mutational patterns. Sub-clonal analysis was conducted by adding "-u" parameter to input commands.

Analysis of RNA sequencing
Paired end sequencing reads of cDNA libraries (101bp) generated from a NovaSeq6000 instrument were verified its sequence quality with FastQC v 0.11.7. For data preprocessing, low quality bases and adapter sequences in reads were trimmed using Trimmomatic v 0.38. The trimmed reads were aligned to the human genome (UCSC hg19) using HISAT v2.1.0, a splice-aware aligner. And then, transcript assembly of known transcripts, novel transcripts, and alternative splicing transcripts was processed by StringTie v1.3.4d. Based on the result of that, expressional abundance of transcript and gene were calculated as read count or TPM value (Transcript per Million mapped reads) per sample.

Analysis of Methylation sequencing
The capture libraries for targeted methylation sequencing were prepared according to the manufacturer's instructions of SureSelectXT Methyl-Seq Library Preparation kit. (Agilent Technologies, Germany). Briefly, Fragmentation of 3ug of genomic DNA was performed using the Covaris LE220 focused-ultrasonicator (Covaris, Woburn, MA) to a target peak size of 150–200 bp. Load the 8 microTUBE Strip into the tube holder of the ultrasonicator and shear the DNA using the following settings: mode, frequency sweeping; duty cycle, 10%; intensity, 5; cycles per burst, 200; duration, 60 sec × 6 cycles; temperature, 4–7°C. The fragmented DNA is repaired, an 'A' is ligated to the 3' end, SureSelect Methyl-Seq Methylated Adapter are then ligated to the fragments. The methylated adapter-ligated DNA is then quantified using the TapeStation DNA screentape D1000 (Agilent). For methyl-C sequence capture, 350 ng of DNA library was mixed with hybridization buffers, blocking mixes, RNase block and 5 µl of SureSelect Methyl-Seq Capture Library, according to the standard Agilent SureSelectXT Methyl-Seq Target Enrichment protocol. Hybridization to the capture baits was conducted at 65°C using heated thermal cycler lid option at 105°C for 24 hours on PCR machine. The captured DNA was then washing and amplified. The final purified product is then quantified using qPCR according to the qPCR Quantification Protocol Guide Guide (KAPA Library Quantificatoin kits for Illumina Sequecing platforms) and qualified using the TapeStation DNA screentape D1000 (Agilent). And then we sequenced using the HiSeq platform (Illumina,San Diego, USA).

Multi-omics integration
The drug response data is integrated with RNA-seq and methyl-seq data using mixOmics R Bioconductor package with build-in analyzing and visualization functions. We have limited the construction matrix with 3 parameters: RNA-seq (759) x methyl-seq (328) x drug responses (25) estimating more than six million multi-omics combinations. We selected the expressional patterns and promotor methylation status of tumorigenesis-related genes by referring previously reported pan-cancer driver genes. Detailed code is described in the code availability section.

For manuscripts utilizing custom algorithms or software that are central to the research but not yet described in published literature, software must be made available to editors and reviewers. We strongly encourage code deposition in a community repository (e.g. GitHub). See the Nature Portfolio guidelines for submitting code & software for further information.

Data

Policy information about availability of data

All manuscripts must include a data availability statement. This statement should provide the following information, where applicable:
- Accession codes, unique identifiers, or web links for publicly available datasets
- A description of any restrictions on data availability
- For clinical datasets or third party data, please ensure that the statement adheres to our policy

Raw and processed next-generation sequencing data, in accordance with the MINSEQE standards will be deposited in a public database and will be freely available. Few processed sequencing data will be available by request and governed by the corresponding authors. Computational pipelines as well as sequencing data including whole exome sequencing and RNA-sequencing in this study are available at the public repository (https://github.com/nahyun828/SIC).

Field-specific reporting

Please select the one below that is the best fit for your research. If you are not sure, read the appropriate sections before making your selection.

☒ Life sciences ☐ Behavioural & social sciences ☐ Ecological, evolutionary & environmental sciences

For a reference copy of the document with all sections, see nature.com/documents/nr-reporting-summary-flat.pdf

Life sciences study design

All studies must disclose on these points even when the disclosure is negative.

Sample size	One patient with six different tumors along bowel tract.
Data exclusions	No data were excluded from the analysis.
Replication	The reproducibility of the drug library screening was confirmed by triplicating the entire experiment procedure. All attempts at replication were successful.
Randomization	This study includes a single participant, and there was no randomization.
Blinding	This study includes a single participant, and there was no group allocation during data collection and/or analysis.

Reporting for specific materials, systems and methods

We require information from authors about some types of materials, experimental systems and methods used in many studies. Here, indicate whether each material, system or method listed is relevant to your study. If you are not sure if a list item applies to your research, read the appropriate section before selecting a response.

Materials & experimental systems

n/a	Involved in the study	
☐	☒	Antibodies
☐	☒	Eukaryotic cell lines
☒	☐	Palaeontology and archaeology
☐	☒	Animals and other organisms
☐	☒	Human research participants
☐	☒	Clinical data
☒	☐	Dual use research of concern

Methods

n/a	Involved in the study	
☒	☐	ChIP-seq
☒	☐	Flow cytometry
☒	☐	MRI-based neuroimaging

Antibodies

Antibodies used	Mouse monoclonal anti-Cytokeratin 20 Santa Cruz Biotechnology Cat# sc-271183; RRID:AB_10610054 1:200<br>Mouse monoclonal anti-CDX2 BioGenex Cat# AM392; RRID:AB_2650531 1:300<br>Mouse monoclonal anti-β-catenin BD Biosciences Cat# 610153; RRID:AB_397554 1:800<br>Rabbit polyclonal anti-KI-67 Abcam Cat# ab15580; RRID:AB_443209 1:500<br>Mouse monoclonal anti-MLH1 Santa Cruz Biotechnology Cat# sc-56161; RRID:AB_784582 1:500<br>Mouse monoclonal anti-MSH2 Santa Cruz Biotechnology Cat# sc-137015; RRID:AB_2144968 1:500<br>Mouse monoclonal anti-MSH6 Santa Cruz Biotechnology Cat# sc-271080; RRID:AB_10611658 1:500<br>Mouse monoclonal anti-PMS2 Santa Cruz Biotechnology Cat# sc-25315; RRID:AB_628163 1:500
Validation	All antibodies suit for the immunohistochemistry of colon normal and tumor tissue.

Eukaryotic cell lines

Policy information about cell lines

Cell line source(s)	We established patient-derived organoids (PDOs), cell lines (PDCs), and organoid cell lines (PDOC) from each of six different sites of one synchronous intestinal tumor, resulting in 18 preclinical in vitro models.
Authentication	Short tandem repeat (STR) profiling validated that all derivatives shared matched loci without cross-contamination.
Mycoplasma contamination	All in vitro models tested negative for mycoplasma contamination.
Commonly misidentified lines (See ICLAC register)	N/A

Animals and other organisms

Policy information about studies involving animals; ARRIVE guidelines recommended for reporting animal research

Laboratory animals	Mouse, BALB/c nude, Male, 8 weeks

Wild animals	The study did not involve wild animals.
Field-collected samples	The study did not involve samples collected from the field.
Ethics oversight	The research protocol was reviewed and approved by the institutional review board of the Korea Research Institute of Bioscience and Biotechnology (IRB No. KRIBB-AEC-2118).

Note that full information on the approval of the study protocol must also be provided in the manuscript.

Human research participants

Policy information about studies involving human research participants

Population characteristics	Age: Male, Gender: 61. All other detailed information about the human research participant is described in Table 1.
Recruitment	A patient with pathologically proven synchronous intestinal carcinoma was recruited by hospital staff.
Ethics oversight	The research protocol was reviewed and approved by the institutional review board of the Seoul National University Hospital (IRB No. 1102-098-357).

Note that full information on the approval of the study protocol must also be provided in the manuscript.

Clinical data

Policy information about clinical studies

All manuscripts should comply with the ICMJE guidelines for publication of clinical research and a completed CONSORT checklist must be included with all submissions.

Clinical trial registration	We have not registered this pre-pilot study yet, but plan to register the larger pilot study required for an FDA submission.
Study protocol	Study protocol is available by emailing to the corresponding author (kujalok@snu.ac.kr).
Data collection	The sample and data were collected by the corresponding author (minjungkim@snuh.org).
Outcomes	Primary outcome measure was the cancer diagnosis based on histopathology reports.
